# Honokiol enhances the sensitivity of cetuximab in KRAS^G13D^ mutant colorectal cancer through destroying SNX3-retromer complex

**DOI:** 10.7150/thno.97180

**Published:** 2024-08-26

**Authors:** Qianru Zhu, Ruonan Zhang, Xiaoqing Gu, Ziming Zhao, Quan Gao, Min Chen, Qibiao Wu, Tian Xie, Xinbing Sui

**Affiliations:** 1 School of Pharmacy, Hangzhou Normal University, Hangzhou, 311121, Zhejiang, China.; 2 State Key Laboratory of Quality Research in Chinese Medicines, Faculty of Chinese Medicine, Macau University of Science and Technology, Macau, P.R. China.; 3 Department of Medical Oncology, the Affiliated Hospital of Hangzhou Normal University, Hangzhou Normal University, Hangzhou, Zhejiang 311121, China.

**Keywords:** colorectal cancer, honokiol, cetuximab, KRAS, SNX3

## Abstract

***Rationale***: the proto-oncogene *KRAS* is frequently mutated in colorectal cancer (CRC), leading to inherent resistance against monoclonal antibodies targeting the epidermal growth factor receptor (EGFR), such as cetuximab. Therefore, addressing the primary resistance and expanding the indications for target therapy have become critical challenges.

***Methods***: the screening of a natural product library against KRAS mutant CRC cells was conducted, leading to the discovery of a small molecule compound that sensitive to the KRAS^G13D^ mutation site. The anti-tumor activity of this small molecule compound in combination with cetuximab was evaluated using the KRAS^G13D^ mutant CRC models both *in vivo* and *in vitro*. This evaluation includes an examination of its effects on cell proliferation, viability, apoptosis, cell cycle progression, and tumor growth. Furthermore, RNA sequencing, western blot analysis, immunofluorescence, real-time quantitative PCR, and pull-down assays were employed to explore the molecular mechanisms underlying the synergistic anti-tumor effect of this small molecule compound in combination with cetuximab.

***Results***: our study screened 882 compounds in KRAS mutant CRC cells and identified honokiol, a small molecule compound that exhibits specific sensitivity to KRAS^G13D^ mutant CRC cells. Furthermore, we revealed that the synergistic augmentation of cetuximab's sensitivity *in vivo* and *in vitro* models of KRAS^G13D^ mutant CRC in combination with honokiol. Mechanistically, honokiol suppresses SNX3-retromer mediated trafficking, thereby impeding lysosomal proteolytic capacity and inhibiting autophagy and macropinocytosis fluxes. Moreover, honokiol inhibits the conversion of RAS GDP to RAS GTP, heightening the susceptibility of KRAS^G13D^ CRC mutant cells to cetuximab.

***Conclusions***: honokiol enhances the sensitivity of cetuximab by destroying SNX3 retromer in KRAS^G13D^ mutant CRC preclinical model. These findings present a promising strategy for expanding the indications of target therapy in KRAS mutant colorectal cancer patients.

## Introduction

Kirsten rat sarcoma virus oncogene homologue (*KRAS*) is the most frequently mutated oncogene in human cancer. The KRAS protein regulates cell survival by switching between “on” states with guanosine triphosphate (GTP) binding and “off” states with guanosine diphosphate (GDP) binding. *KRAS* mutations drive oncogenesis in up to 50% of patients with metastatic colorectal cancer (mCRC) 1, with roughly 2/3 of these mutations occurring in codon 12 and 1/3 in codon 13 2. Currently, the primary treatment for mCRC involves fluoropyrimidine-based chemotherapy and targeted therapy 3, 4. Cetuximab, a targeted antibody to Epidermal Growth Factor Receptor (EGFR), has successfully extended overall survival for mCRC patients with wild-type KRAS 5, 6. ​ However, KRAS mutations typically confer primary resistance to cetuximab due to the uncontrolled activation of the KRAS signaling cascade. Among the various KRAS mutations, the G13D variant behaves differently. Previous studies have reported that CRC patients with KRAS^G13D^ mutation may exhibit sensitivity to EGFR inhibitors 7, 8. This sensitivity is attributed to the tumor suppressor NF1 binding to KRAS^G13D^, thereby stimulating the hydrolysis of GTP 9, 10. Despite this limited sensitivity to cetuximab, KRAS^G13D^ mutations are still associated with a poor prognosis, and the mechanisms underlying this primary resistance are not well understood. Therefore, overcoming the primary resistance of KRAS^G13D^, augmenting the anticancer efficacy of cetuximab, and expanding the indications for cetuximab have become critical challenges.

Currently, combination therapies are attracting more and more attention in many cases and have been developed to augment efficacy, reduce adverse effects and overcome drug resistance for the treatment of cancer patients. Among them, the integration of natural products is increasing. To explore compounds that are sensitive to KRAS^G13D^ mutation, we established a high-throughput drug screening platform with a natural product compound library. Here, we identify honokiol, a small-molecule compound that is specifically sensitive to KRAS^G13D^ CRC cells. Of note, we observed that the combination of honokiol (HNK) and cetuximab significantly reduced the viability and proliferation of CRC KRAS^G13D^ cells. After an elaborate investigation, the underlying mechanism was elucidated, showing that this combination disrupts the membrane trafficking of the SNX3-retromer, inhibiting the flux of autophagy and macropinocytosis in the KRAS^G13D^ mutant CRC cell line. Simultaneously, the dysfunctional SNX3-retromer inhibits the conversion of RAS GDP to RAS GTP, enhancing the sensitivity of cetuximab and thus amplifying the cytotoxic effects of the combination therapy. These findings provide a promising strategy for CRC patients with primarily resistant to cetuximab therapy.

## Results

### Identification of honokiol as a small molecule compound that is sensitive to KRAS^G13D^ CRC cells

**Figure 1:**
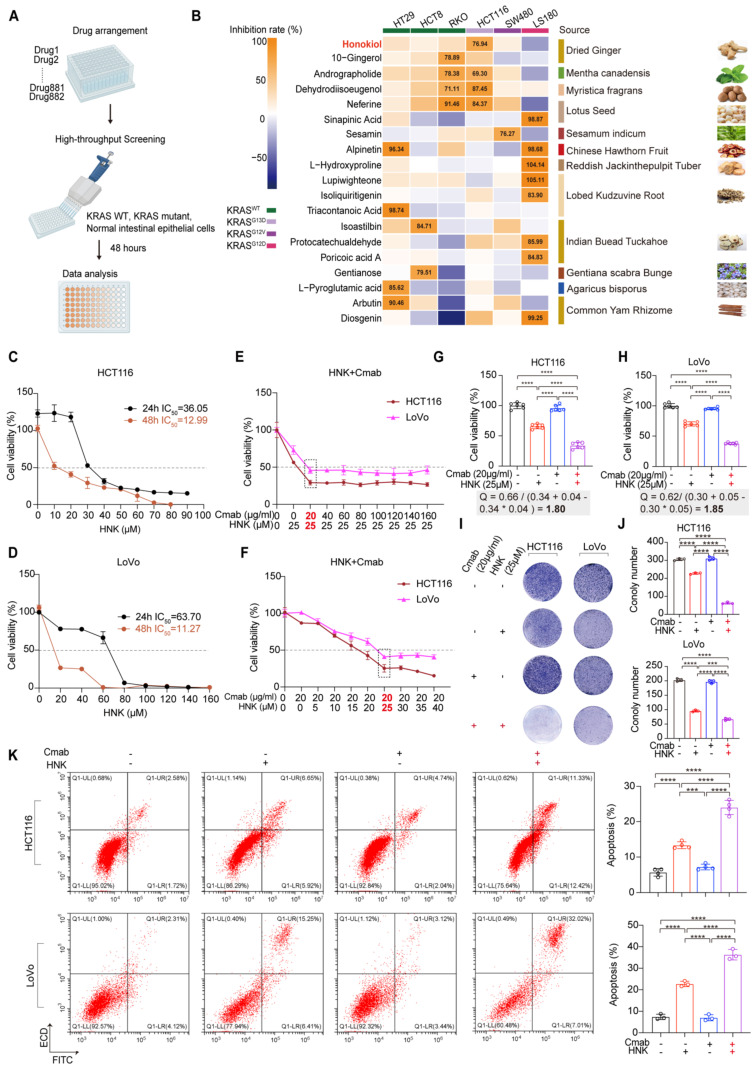
** Identification of honokiol as a small molecule compound that is sensitive to KRAS^G13D^ mutant CRC cell, and low-dose synergistic therapeutic strategy achieved by the combination of honokiol and cetuximab. (A)** Study design. KRAS wild-type and mutant cells treated with individual compounds for drug sensitivity. Figure created with BioRender.com. **(B)** *In vitro* screening of compounds. KRAS wild-type and mutant cells were cultured in the presence of compounds at a single concentration of 20 μM for 48 h. Honokiol was identified as small molecule compound that is sensitive to HCT116 cells. **(C-D)** Percentage of cell viability in HCT116 and LoVo cells after HNK treatment for 24 and 48 h. **(E)** Cell viability by administrating various concentrations of cetuximab (Cmab) and 25 μM HNK in HCT116 and LoVo cells. **(F)** Cell viability by administrating vary concentrations of HNK and 20 μg/ml Cmab in HCT116 and LoVo cells. **(G-H)** Q values of two CRC cell lines treated with Cmab alone (20 μg/ml), HNK alone (25 μM), or the combination of Cmab (20 μg/ml) and HNK (25 μM). Jin's formula: Q = E(a+b) / (Ea+Eb - Ea*Eb). Ea and Eb represent the cell proliferative inhibition rate for individual drug; E(a+b) represents the cell proliferative inhibition rate for combined drug. Q value indicates a synergistic effect. Q < 0.85 indicates antagonism; 0.85 ≤ Q < 1.15 indicates additive effects; Q ≥ 1.15 indicates synergism. **(I-J)** Colony forming of Cmab and HNK treatment. Colony formation was fixed in methanol, stained with crystal violet, and counted. **(K)** Apoptosis rate was assessed by flow cytometry after staining with annexin V-FITC/PI in HCT116 and LoVo cells following HNK and Cmab treatment for 24 h. Data are represented as mean ± S.D. from 3 independent experiments. *p < 0.05, **p < 0.01, ***p < 0.001, and ****p < 0.0001 by one-way ANOVA test with Tukey multiple comparisons.

To identify clinical therapeutic compounds active in KRAS mutant CRC cells, we performed an *in vitro* screening using a customized natural product library to assess drug sensitivity in KRAS wild-type cells (HT29, HCT8, RKO), KRAS^G12D^ (LS180), KRAS^G12V^ (SW480), and KRAS^G13D^ (HCT116) mutant cells (**Figure 1A**). Cells were cultured in the presence of compounds at a single concentration of 20 μM for 48 hours, and viability was quantified by CCK8 assay. Our initial screen identified 339 compounds that did not inhibit the viability of normal intestinal epithelial cells (NCM460). Subsequently, 20 candidate compounds exhibited a greater than 75% inhibition rate in at least one CRC cell line. Among these, honokiol emerged as the top candidate, demonstrating a prominent inhibitory effect on KRAS^G13D^ mutant CRC cells (**Figure 1B**). Based on these findings, honokiol is considered a potentially therapeutic small molecule, thereby serving as promising candidate for combination therapy with cetuximab against KRAS^G13D^ mutant CRC.

### Low-dose synergistic therapeutic strategy achieved by the combination of honokiol and cetuximab

Next, the cytotoxic effects of HNK were assessed on various cell types, including NCM460, SW480 (KRAS^G12V^), HT29 (KRAS wild-type), HCT116 (KRAS^G13D^), and LoVo (KRAS^G13D^) cells. Cells were treated with escalating doses of HNK treatment for 24 and 48 h, and cell viability was measured by using CCK8 assays. The IC_50_ values of HNK for HCT116, LoVo, SW480, HT29 and NCM460 cells were 36.05, 63.70, 39.88, 38.62, and 43.72 μM, respectively, after 24 h of treatment. After 48 h, the corresponding IC_50_ values decreased to 12.99, 11.27, 26.76, 25.88, and 25.87 μM, respectively (**Figure 1C-D, Figure S1A-C**). These results suggest that the KRAS^G13D^ mutant cell line (HCT116 and LoVo) is the most sensitive to HNK treatment over 48 h.

In addition, NCM460, HT29, SW480, HCT116 and LoVo cells were used for sensitivity testing of cetuximab. The results indicated that cetuximab exhibited moderate sensitivity in KRAS^G13D^ mutant cells compared to KRAS wild-type cells, with the highest resistance observed in KRAS^G12V^ mutant cell line (**Figure S1D-H**).

Furthermore, to evaluate the potential synergistic effects of HNK and cetuximab, cell viability analyses were conducted using various concentrations of cetuximab in combination with 25 μM HNK across different cell types. Co-administration of 25 μM HNK and 20 μg/ml cetuximab did not significantly inhibit the cell viability of NCM460 cells and SW480 cells (**Figure S1I**). Conversely, co-treatment dramatically inhibited cell viability in HCT116, LoVo, and HT29 cells (**Figure 1E, Figure S1I**), suggesting a comparable synergistic effect of HNK and cetuximab toward KRAS^G13D^ mutant and KRAS wild-type cell lines. Intriguingly, the inhibitory effect persisted even with increased HNK concentrations in HCT116 and LoVo cells (**Figure 1F**).

Then, we assessed the synergy index (Q value) of HNK and cetuximab by utilizing Jin's formula, with Q values exceeding 1.15 are indicative of synergism between the two drugs 11. Notably, the Q values of HCT116 and LoVo cells when co-treated with 25 μM HNK and 20 μg/ml cetuximab were 1.80 and 1.85, respectively. These findings indicate a synergistic therapeutic performance with lower doses of HNK and cetuximab in KRAS^G13D^ mutant cell lines (**Figure 1G-H**). In addition, colony formation assays were performed to investigate the proliferation of HCT116 and LoVo cells exposed to HNK and/or cetuximab treatment, and we identified that this combination treatment exhibited a synergistic anti-proliferative effect when compared with cetuximab or HNK treated alone (**Figure 1I-J**). Moreover, annexin V/propidium iodide (PI) staining indicated significant apoptosis following the combination treatment for 24 h (**Figure 1K**). These findings collectively suggest that HNK in combination with cetuximab inhibits cell proliferation, promotes apoptosis, and enhances the cytotoxicity of cetuximab *in vitro*.

### Honokiol enhances the sensitivity of cetuximab by inhibiting autophagic and macropinocytotic fluxes in KRAS^G13D^ mutant CRC cells

**Figure 2:**
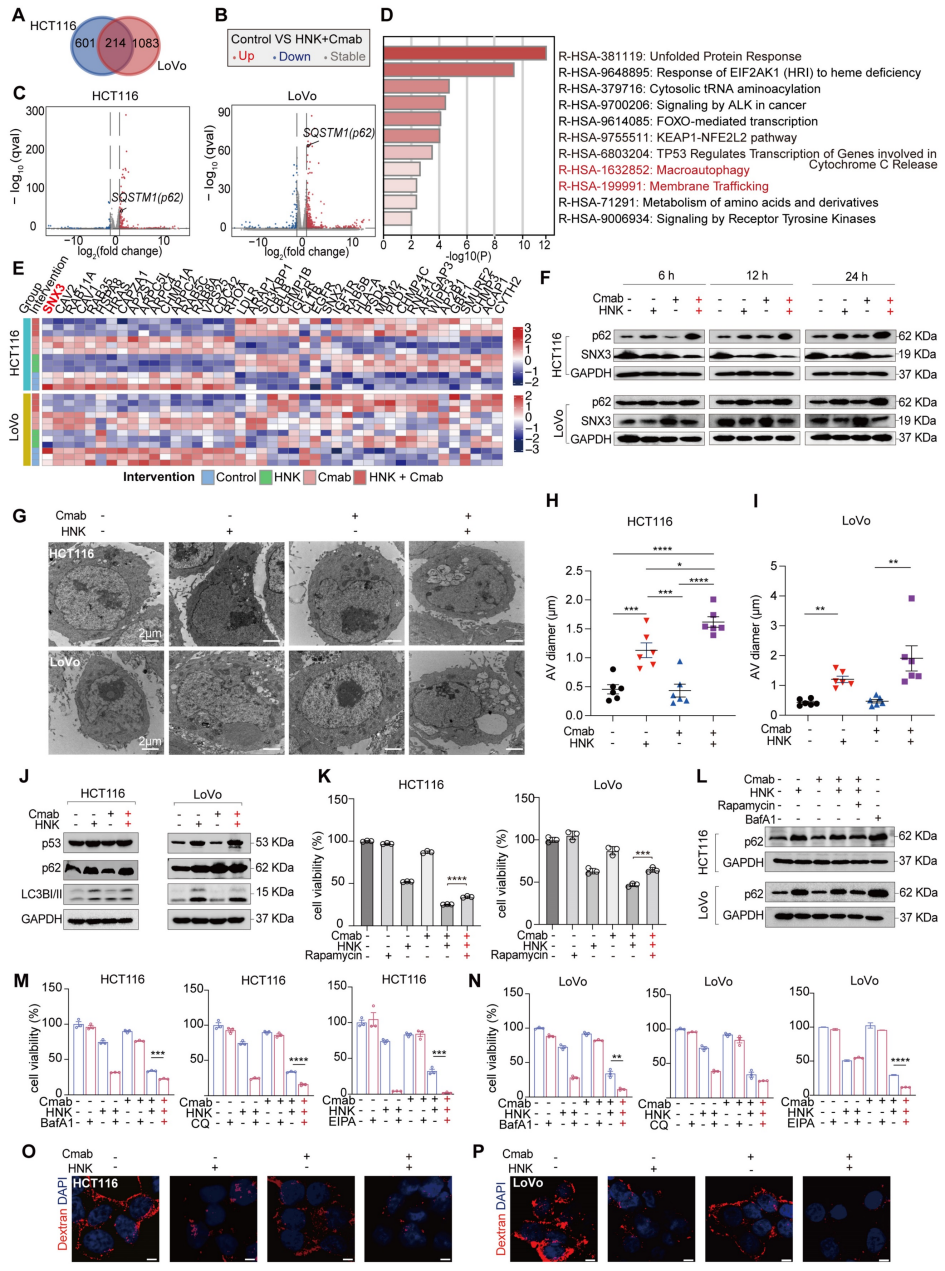
** Honokiol enhances the sensitivity of cetuximab by inhibiting autophagic and macropinocytotic fluxes in KRAS^G13D^ mutant CRC cells. (A)** Venn diagram of HNK + Cmab versus control group in HCT116 and LoVo cells (adjusted p value < 0.05, | log_2_(fold change) | ≥ 0.5). **(B-C)** Volcano plot of HNK + Cmab versus control group in HCT116 and LoVo cells. **(D)** Pathways enrichment based on 214 differentially genes expressed using Metascape (log_10_P-value) in CRC cell lines. (**E**) Heatmap of genes in HCT116 and LoVo cells among control, HNK, Cmab, and HNK + Cmab groups in endocytosis pathway. **(F)** The expressions of p62 and SNX3 proteins at different timepoints after HNK and Cmab combination. **(G)** Representative electron micrographs of HCT116 and LoVo cells treated with HNK and Cmab for 24 h. **(H-I)** Quantification of the size of the multivesicular autophagic vacuoles (AVs) from counts of cell treated with HNK and Cmab. Data are shown as mean ± S.E.M. (n = 5). *p < 0.05, **p < 0.01, ***p < 0.001, and ****p < 0.0001 by one-way ANOVA test with Tukey's multiple comparisons in (H), and Welch ANOVA test with Dunnett's T3 multiple comparisons test in (I). **(J)** Immunoblotting shows that treatment with HNK and Cmab resulted in up-regulation of autophagy markers p62 and LC3-I/II. **(K)** Cell viability of CRC cell lines treated with HNK and Cmab were rescued by rapamycin. ***p < 0.001, and ****p < 0.0001 by unpaired two-tailed t test. **(L)** Immunoblotting of p62 were downregulate after autophagy inducer rapamycin cotreated with HNK and Cmab for 24 h in CRC cell lines. **(M)** Cell viability by administrating BafA1 (50 nM), CQ (20 μM), and EIPA (25 μM) in HCT116 cells, ***p < 0.001, and ****p < 0.0001 by unpaired two-tailed t test. (**N**) Cell viability by administrating BafA1 (50 nM), CQ (5 μM), and EIPA (25 μM) in LoVo cells. **p < 0.01, and ****p < 0.0001 by unpaired two-tailed t test. (**O-P**) Macropinocytosis levels following HNK and Cmab co-treated for 24 h in HCT116 and LoVo cells, scale bar = 5 μm.

In this section, we aim to identify potential pathways and targets involved in the synergistic effects by analyzing gene expression at the transcription level. Of note, a large number of genes were differentially expressed in HCT116 and LoVo cells after being treated by the combination of HNK and cetuximab. The Venn diagram demonstrated that 214 genes were differentially expressed when HNK and cetuximab were combined compared to control in HCT116 and LoVo cells (adjusted p value < 0.05, | log_2_ (fold change) | ≥ 0.5) (**Figure 2A**). Of note, the volcano plot indicated a significant up-regulation of the FPKM of *SQSTM1/p62* in both HCT116 and LoVo cells (**Figure 2B-C**). Subsequent gene ontology analysis of the 214 genes revealed that co-treatment affects the autophagy and membrane trafficking pathways (**Figure 2D**). Moreover, KEGG analysis among control, HNK, cetuximab (Cmab), and HNK + Cmab groups showed significant alternations in the endocytosis pathway in KRAS^G13D^ mutant CRC cells **(Figure S2A-B)**. Interestingly, the heatmap of differentially expressed genes among the four groups indicated that the *SNX3* gene plays a critical role in endocytosis pathway in both HCT116 and LoVo cells (**Figure 2E**). To confirm this finding, we also detected p62 and SNX3 protein levels in HCT116 and LoVo cells. Intriguingly, the combination of HNK and cetuximab elicited a progressive accumulation of p62 protein and a reduction of SNX3 protein in a time-dependent manner (**Figure 2F**). Furthermore, we observed that the accumulation of autophagic vesicles with undigested contents were dramatically increased in both HCT116 and LoVo cells following co-treatment by using transmission electron microscopy analysis (**Figure 2G-I**). Besides, western blot analysis confirmed the upregulation of autophagy markers p62 and LC3I/II, accompanied by elevated expression of p53 in KRAS^G13D^ mutant CRC cells (**Figure 2J**). Notably, autophagy inducer (rapamycin) restored the cell viability of the HCT116 and LoVo cells (**Figure 2K**) and mitigated the upregulation of p62 protein in the presence of HNK and cetuximab co-treatment (**Figure 2L**), Conversely, using BafA1, CQ (autophagy inhibitors), and EIPA (macropinocytosis inhibitor) significantly increased lethality in HCT116 cells after co-treatment (**Figure 2M**). Uniformly, BafA1 and EIPA had a significant inhibitory effect, while CQ showed a similar trend in LoVo cells, although the difference was not significant in LoVo cells (**Figure 2N**). Meanwhile, there was a reduction in macropinocytosis levels following honokiol and cetuximab co-treated in CRC cells (**Figure 2O-P).** Collectively, these data suggest that the inhibition of autophagy and macropinocytosis fluxes contributes to cytotoxicity of KRAS^G13D^ mutant CRC cells in the presence of cetuximab combined with HNK.

### Honokiol increases the cytotoxicity of KRAS^G13D^ mutant CRC cells to cetuximab through disrupting SNX3-retromer

The induction of autophagy and macropinocytosis affected by aberrant retromer trafficking, has previously been reported in Hela cells. Similarly, we observed alterations in membrane trafficking and corresponding reductions of SNX3, suggesting that this gene plays a crucial role in the combination of honokiol and cetuximab (**Figure 2E**).

**Figure 3:**
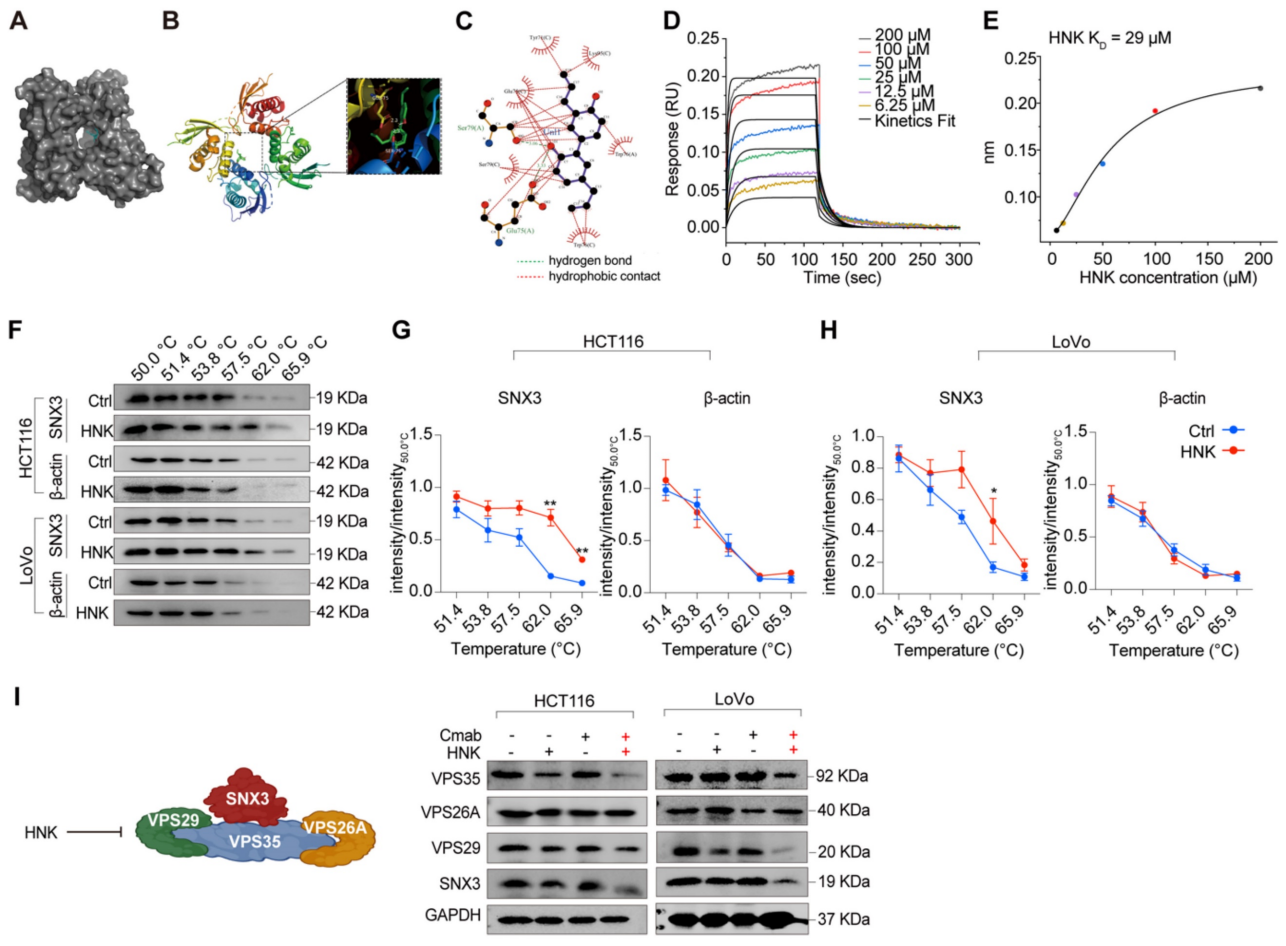
** Honokiol targets the SNX3 protein. (A-C)** The results of molecular docking between SNX3 protein and HNK. **(D-E)** Real-time kinetic binding parameter of HNK interacting with SNX3 based on biolayer interferometry.** (F)** CETSA was performed in HCT116 and LoVo cells. Cell lysates in control group (0.1% (v/v) DMSO) and HNK treatment group were subjected to western blot analysis. **(G-H)** CETSA curves of SNX3 or beta-actin in HCT116 and LoVo cells were determined with 0.1% (v/v) DMSO and 25 μM HNK treatment. Normalized band intensity ratios from three independent experiments are presented as mean ± s.e.m, *p < 0.05, **p < 0.01 by unpaired two-tailed t test. **(I)** Immunoblotting of SNX3, VPS26A, VPS29, and VPS35 in CRC cell lines after HNK and Cmab treated for 24 h.

Furthermore, molecular docking results suggest that honokiol may bind between two SNX3 proteins, with a hydrogen bond to Ser79 or Glu75, and hydrophobic contacts with Tyr71, Glu75, Trp76, Ser79, and Lys95 (**Figure 3A-C**). To further confirm the direct interaction between honokiol and SNX3 protein, biolayer interferometry analysis was performed to determine the dissociation constant (K_D_). Firstly, the real-time binding/dissociation of HNK with SNX3 was monitored by increasing the concentration of HNK (0, 6.25, 12.5, 25, 50, 100, and 200 μM) to examine the binding effect of HNK with SNX3. The results show that the curve of association and dissociation between HNK and SNX3, as well as their steady-state curves with a K_D_ value of 29 μM verified the direct binding between them (**Figure 3D-E**). In addition, we performed cellular thermal shift assays to identify binding ability of HNK and SNX3 protein, and we found that HNK binds to SNX3 protein more strongly than its control (**Figure 3F-H**). To further determine retromer function after combination treatment, we investigated the expression of SNX3 subunits at mRNA and protein levels. As expected, we observed that the relative expression of *SNX3* gene was significantly decreased in LoVo cells after honokiol and cetuximab co-treatment, while it remained stable in HCT116 cells. Besides, the relative expression of the trimeric retromer complex (*Vps35, Vps26A* and *Vps29*) was decreased in HCT116 and LoVo cells at the mRNA levels in the combination of honokiol and cetuximab (**Figure S3A-B**). Meanwhile, Vps35, VPS29 and SNX3 protein levels were decreased while VPS26A remained stable following honokiol and cetuximab treatment (**Figure 3I**). Taken together, these data demonstrate that HNK combined cetuximab induces the impairment of SNX3-retromer.

**Figure 4:**
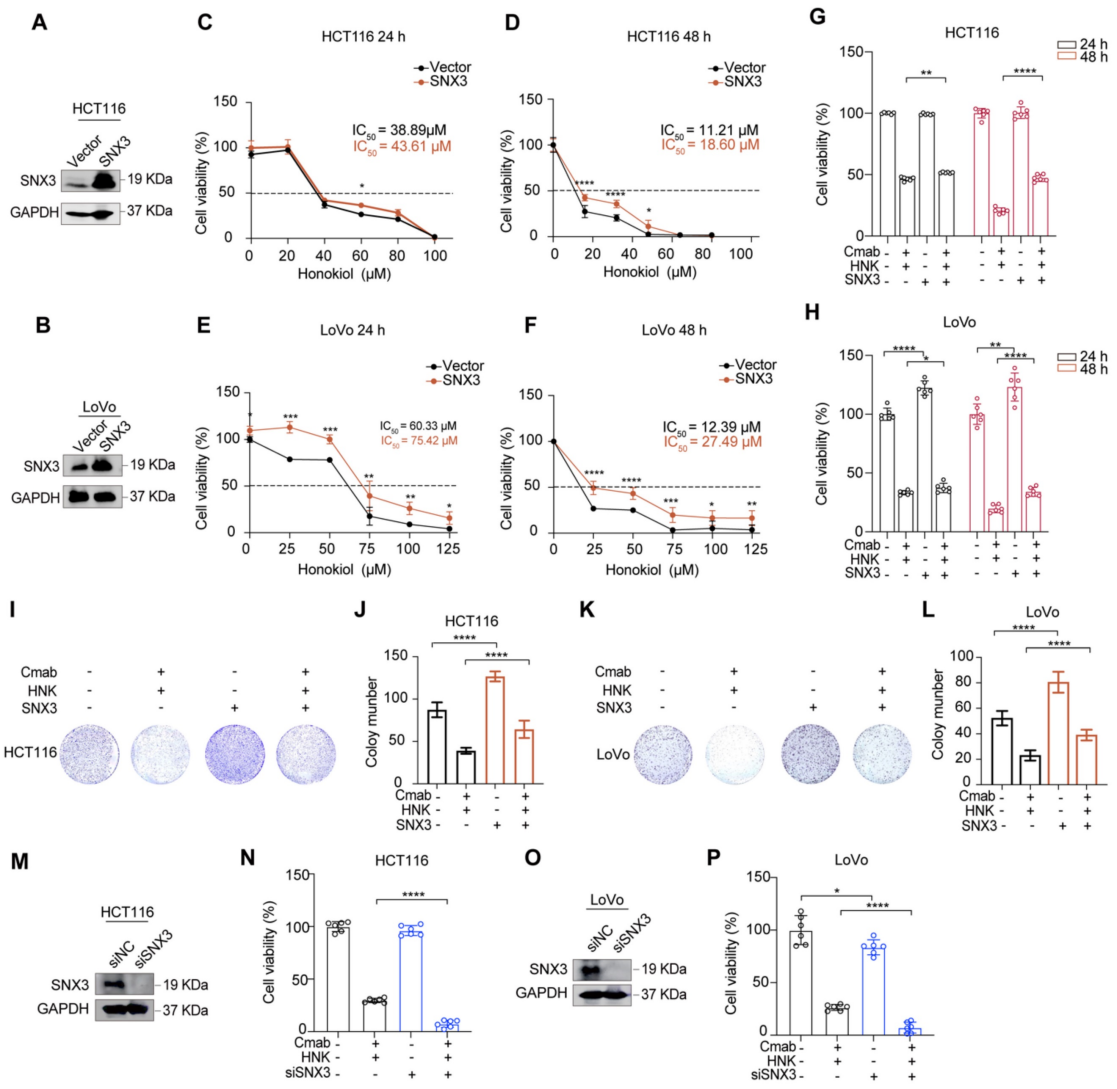
** Honokiol increases the cytotoxicity of KRAS^G13D^ mutant CRC cells to cetuximab through disrupting SNX3 protein. (A-B)** western blot analysis of SNX3 protein in stable over-expressed HCT116 and LoVo cells. **(C-F)** CCK8 analysis was performed to investigate the cell viability of HCT116 and LoVo subjected to stable over-expressed of SNX3 with the indicated doses of honokiol treatment for 24 h and 48 h, *p < 0.05, **p < 0.01, ***p < 0.001, and ****p < 0.0001 by unpaired two-tailed t test. Data are summarized as mean ± S.D. (n = 6). **(G-H)** The cell viability assays were used to analyze the responses of HCT116 and LoVo subjected to the stable over-expressed of SNX3 to the co-treatment (25 μM HNK + 20 μg/ml Cmab) compared to vector groups for 24 h and 48 h. *p < 0.05, **p < 0.01, ***p < 0.001, and ****p < 0.0001 by unpaired two-tailed t test. Data are summarized as mean ± S.D. **(I-L)** The cell proliferation effects of HCT116 (I-J) and LoVo (K-L) over-expressed SNX3 cells with HNK and Cmab treatment were assessed by colony-formation assay. Data are summarized as mean ± S.D., **** p < 0.0001 by unpaired two-tailed t test. (n = 3). **(M, O)** western blot analysis of the effect of SNX3 siRNA in HCT116 and LoVo cells. **(N, P)** CCK8 assays were used to analyze the responses of HCT116 and LoVo cells subjected to the knockdown of SNX3 to the combination treatment (25 μM HNK + 20 μg/ml Cmab) for 24 h compared to control. Data are summarized as mean ± S.D., *p < 0.05, **** p < 0.0001 by unpaired two-tailed t test.

To investigate the role of SNX3 in tumor progression *in vitro*, we constructed stable cell lines with SNX3 over-expression by lentiviral infection. As shown in Figure 4A-B, the expressions of SNX3 were increased at the protein levels in both HCT116 and LoVo cells (**Figure 4A-B**). Subsequently, we identified that SNX3 over-expression significantly increased the IC_50_ values of HNK in KRAS^G13D^ mutant CRC cell lines (**Figure 4C-F**). Moreover, SNX3 over-expression gradually reduced the cytotoxicity of KRAS^G13D^ mutant CRC cells in the combination of HNK and cetuximab (**Figure 4G-H**). Besides, using colony formation assay demonstrated that SNX3 over-expression significantly reduced the inhibitory effects of combination treatment in HCT116 and LoVo cells (**Figure 4I-L**). Conversely, we found that the cell viability was significantly decreased in HCT116 and LoVo cells in the combination of HNK and cetuximab after SNX3 siRNAs transfection for 48 h (**Figure 4M-P**). These data suggest that SNX3 over-expression promotes cell viability and proliferation, while SNX3 knock down suppresses cell viability in HCT116 and LoVo cells in the combination of HNK and cetuximab. Altogether, our data indicate that honokiol increases the cytotoxicity of KRAS^G13D^ mutant CRC cells to cetuximab by disrupting SNX3-retromer.

### SNX3 retromer deficiency alters lysosomal proteolysis

Considering the impairment of the SNX3 retromer in the combination treatment, we wondered whether it affects the function of lysosomes in KRAS^G13D^ mutant cells. To assess this, we treated HCT116 and LoVo cells with lysotracker red, a fluorescent dye that labels acidic endolysosomal compartments. The results demonstrate a reduction of lysotracker-positive organelles after honokiol and cetuximab combination (**Figure S3C**).

**Figure 5:**
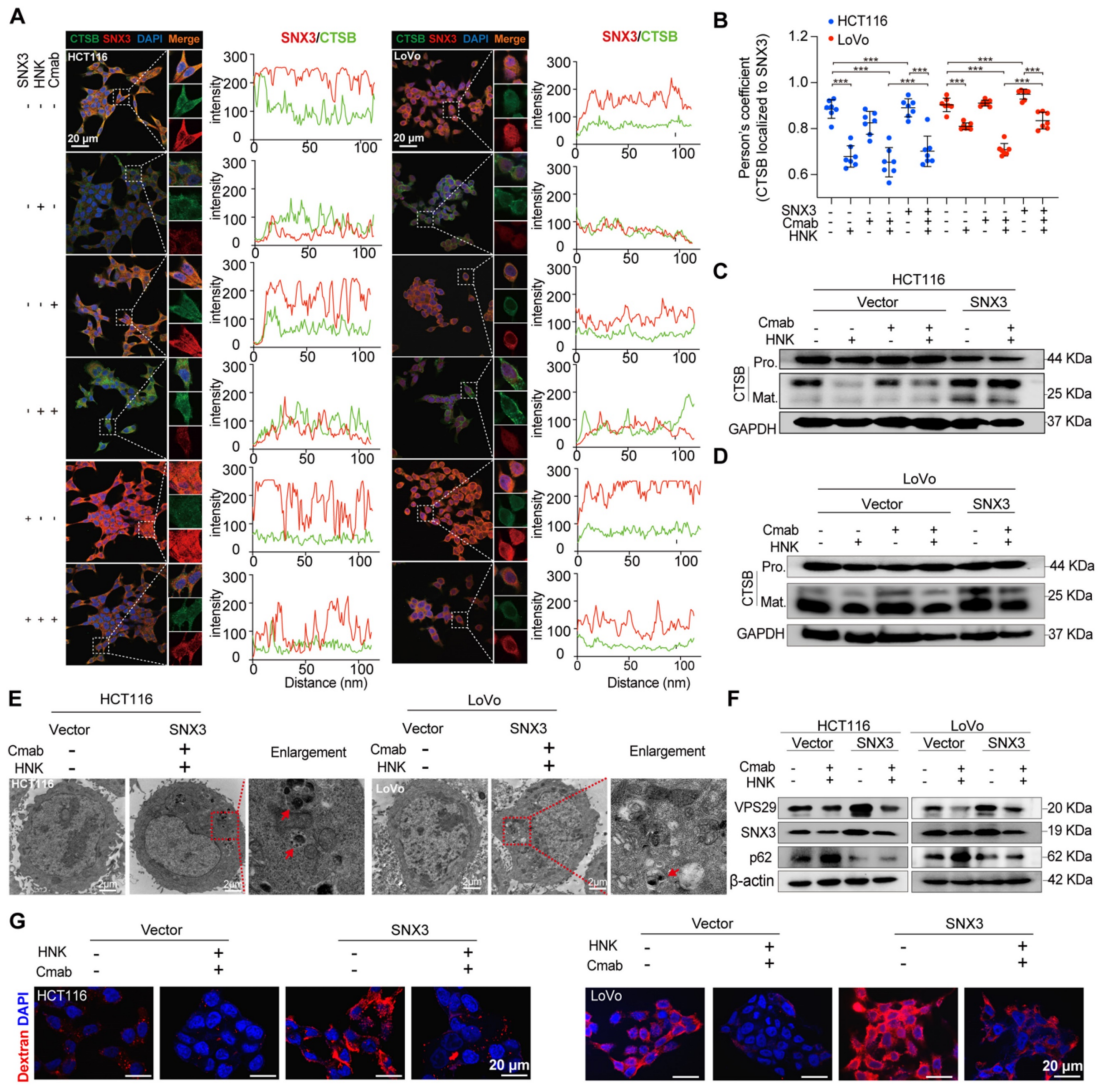
** SNX3 retromer deficiency alters lysosomal proteolysis. (A)** HCT116 and LoVo cells were over-expressed SNX3 and immunolabeled with antibodies against SNX3 and CTSB by Alexa Fluor-conjugated fluorescence secondary antibodies. The intensity plots of the fluorescent intensity (y-axis) against distance (x-axis) represent the overlap between channels. **(B)** The colocalization between SNX3 and CTSB proteins was quantified by Pearson's correlation coefficient (n = 7, mean ± S.D.). One-way ANOVA with Tukey's multiple comparisons test was used to determine the statistical significance. *p<0.05, **p<0.01, ***p<0.001, **** p<0.0001. **(C-D)** Immunoblotting of pro-mature and mature forms of CTSB in SNX3 stable over-expressed CRC cells after the combination of HNK and Cmab for 24 h. **(E)** Representative electron micrographs of SNX3 over-expressed cells treated with HNK and Cmab for 24 h. Arrows means clear vacuoles within the cells infused with lysosomes. **(F)** Immunoblotting of VPS29, SNX3, and p62 in SNX3 over-expressed cells after the combination of HNK and Cmab for 24 h in SNX3 over-expressed cells. **(G)** Macropinocytosis levels following HNK and Cmab co-treated for 24 h in SNX3 over-expression cells.

How does dysfunctional retromer disrupt the function of lysosome? Evidence shows that cathepsin B (CTSB), a lysosomal enzyme, is synthesized as immature proenzyme and subsequently activated through proteolytic processing upon reaching the lysosome 12. Additionally, previous study has shown that retromer dysfunction is associated with the improper processing and secretion of CTSB 13. Besides, retromer is thought to be responsible for the altered retrieval of the cation-independent mannose 6-phosphate receptor (CI-MPR), a receptor that targets hydrolases to the lysosome 14, 15. To ascertain whether the dysfunctional retromer impacts lysosomal enzyme activities, we conducted confocal laser scanning microscopy (CLSM) analysis to determine the spatial distribution of SNX3 and CTSB in HCT116 and LoVo cells following the combination of HNK and cetuximab. CLSM images revealed that the expression of SNX3 was decreased in honokiol treated cells. Interestingly, CTSB expression was not significantly altered in either HCT116 or LoVo cells, regardless of the drug treatment administered (**Figure 5A**).

Intriguingly, colocalization analysis indicated a significant overlap of SNX3 and CTSB in both HCT116 and LoVo cells, as well as in cetuximab-treated HCT116 and LoVo cells (HCT116: R_SNX3-CTSB_= 0.8915, HCT116-Cmab: R_SNX3-CTSB_= 0.8664, LoVo: R_SNX3-CTSB_= 0.9257, LoVo-Cmab: R_SNX3-CTSB_= 0.9070). However, this overlap was disrupted by HNK treatment (HCT116 HNK: R_SNX3-CTSB_= 0.6580, HCT116-Cmab+HNK: R_SNX3-CTSB_= 0.6208, LoVo HNK: R_SNX3-CTSB_= 0.7976, LoVo-Cmab+HNK: R_SNX3-CTSB_= 0.7047), indicating that retromer deficiency disrupts the transport of CTSB to lysosomes in HCT116 and LoVo cells after honokiol treatment. Furthermore, CLSM assay was performed on SNX3 over-expressed cells to determine whether SNX3 impairment affects CTSB translocation. Notably, a slightly improved overlap between SNX3 and CTSB was observed in SNX3 over-expressed KRAS^G13D^ mutant cells treated with HNK and cetuximab (**Figure 5A-B**). Simultaneously, we also identified a significant positive correlation (R = 0.35, p < 0.0001) between *SNX3* and *CTSB* genes in the CRC cohort from the TCGA database (**Figure S4**). Taken together, these data suggest that HNK downregulates the expression of SNX3, thereby perturbing the transport of CTSB to lysosomes.

In addition, we performed western blot analysis to investigate stepwise proteolytic maturation of CTSB. Our findings reveal a dramatic reduction in the mature form of CTSB in HNK treated cells, suggesting that proteolytic formation and maturation are perturbed by HNK treatment (**Figure 5C-D**). These data demonstrate that lysosomal proteolysis is disrupted by the combination of HNK and cetuximab through the downregulation of SNX3 expression.

Next, we questioned whether autophagic and macropinocytotic fluxes were improved in the presence of SNX3 over-expression in CRC cells after the combination therapy. Using transmission electron microscopy, we observed a reduction in autophagic vesicles with fused lysosomes, indicating unobstructed late-stage autophagy in HCT116 and LoVo cells (**Figure 5E**). Moreover, over-expressing SNX3 enhanced the expression of VPS29, accompanied by a reduction of p62 in protein levels, compared to vector-transfected HCT116 and LoVo cells following combination treatment (**Figure 5F**). We subsequently examined the macropinocytosis activity in SNX3 over-expressed KRAS^G13D^ mutant cells. As expected, HCT116 and LoVo cells exhibited higher levels of macropinocytosis than their vehicle-treated counterparts and were able to counteract inhibition by therapeutic drugs (**Figure 5G**). These data indicate that the over-expression of SNX3 improves lysosomal proteolysis, which is essential for the digestion of autophagosomes and macropinosomes.

### Honokiol co-treated with cetuximab inhibits the activation of EGFR signaling cascade

**Figure 6:**
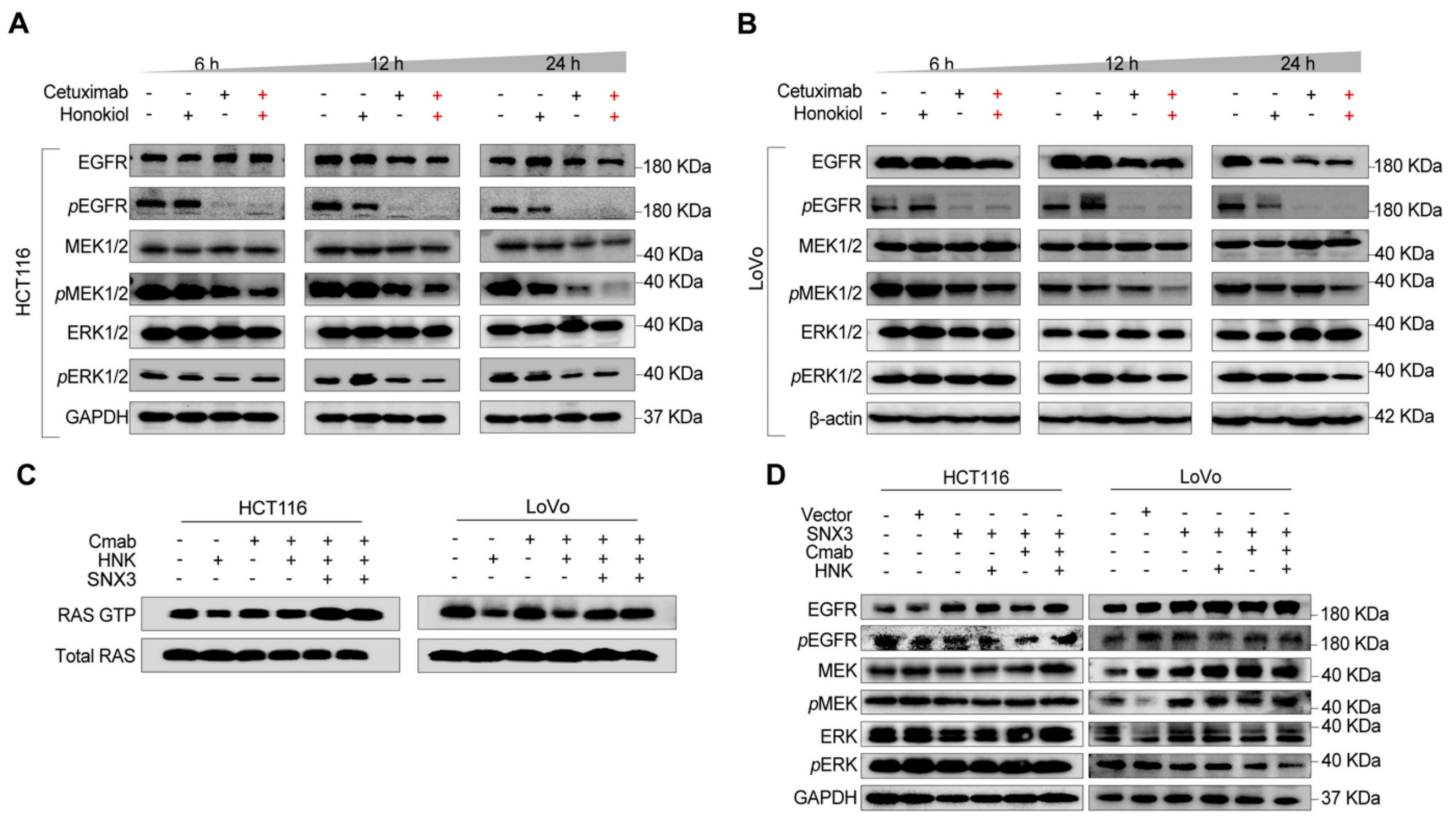
** Honokiol co-treated with cetuximab inhibits the activation of the EGFR signaling cascade. (A-B)** EGFR signaling analysis in CRC cell lines following HNK and Cmab combination at 6, 12, and 24 h by using western blot analysis. **(C)** RAS pull down assay following HNK and Cmab treatment in HCT116 and LoVo cells.** (D)** EGFR cascade analysis of stable over-expressed HCT116 and LoVo cells incubated with HNK and Cmab combination.

In this section, to determine the change of EGFR signaling cascade, phospho-EGFR (*p*EGFR), *p*MEK, and *p*ERK were detected in HCT116 and LoVo cells following HNK and cetuximab treated for 6, 12, and 24 h. We found that *p*EGFR was nearly completely suppressed in both HCT116 and LoVo cells after 6 hours of cetuximab treatment, with a gradual suppression of *p*MEK and *p*ERK following HNK and cetuximab in a time-dependent manner (**Figure 6A-B, Figure S5A-B**). Since sustained RAS activation is a stumbling block for EGFR-targeting inhibitors, we therefore wondered whether HNK could synergistically assist cetuximab by inhibiting RAS activation in KRAS^G13D^ mutant cells. Therefore, we performed a RAS GTP pull-down assay to determine the conversion of RAS GTP and RAS GDP in the combination of HNK and cetuximab. Notably, the conversion of RAS GDP to RAS GTP was inhibited in HNK treated cells and this phenomenon can be reversed by SNX3 over-expression (**Figure 6C**). These results suggest that the EGFR-RAS-MEK-ERK signaling cascade is suppressed in KRAS^G13D^ mutant CRC cells after treatment with HNK and cetuximab.

Furthermore, western blot assay was performed on SNX3 over-expressed cells to identify the change of EGFR signaling pathway in the treatment of HNK and cetuximab, and we observed the upregulation of *p*EGFR, *p*MEK, and *p*ERK in both HCT116 and LoVo cells (**Figure 6D**). Collectively, these results indicate that EGFR-RAS-MEK-ERK pathway is suppressed after cetuximab and honokiol treatment, and this inhibition could be reversed by SNX3 over-expression.

### Honokiol enhances the therapeutic efficacy of cetuximab in KRAS^G13D^ mutant CRC model

**Figure 7:**
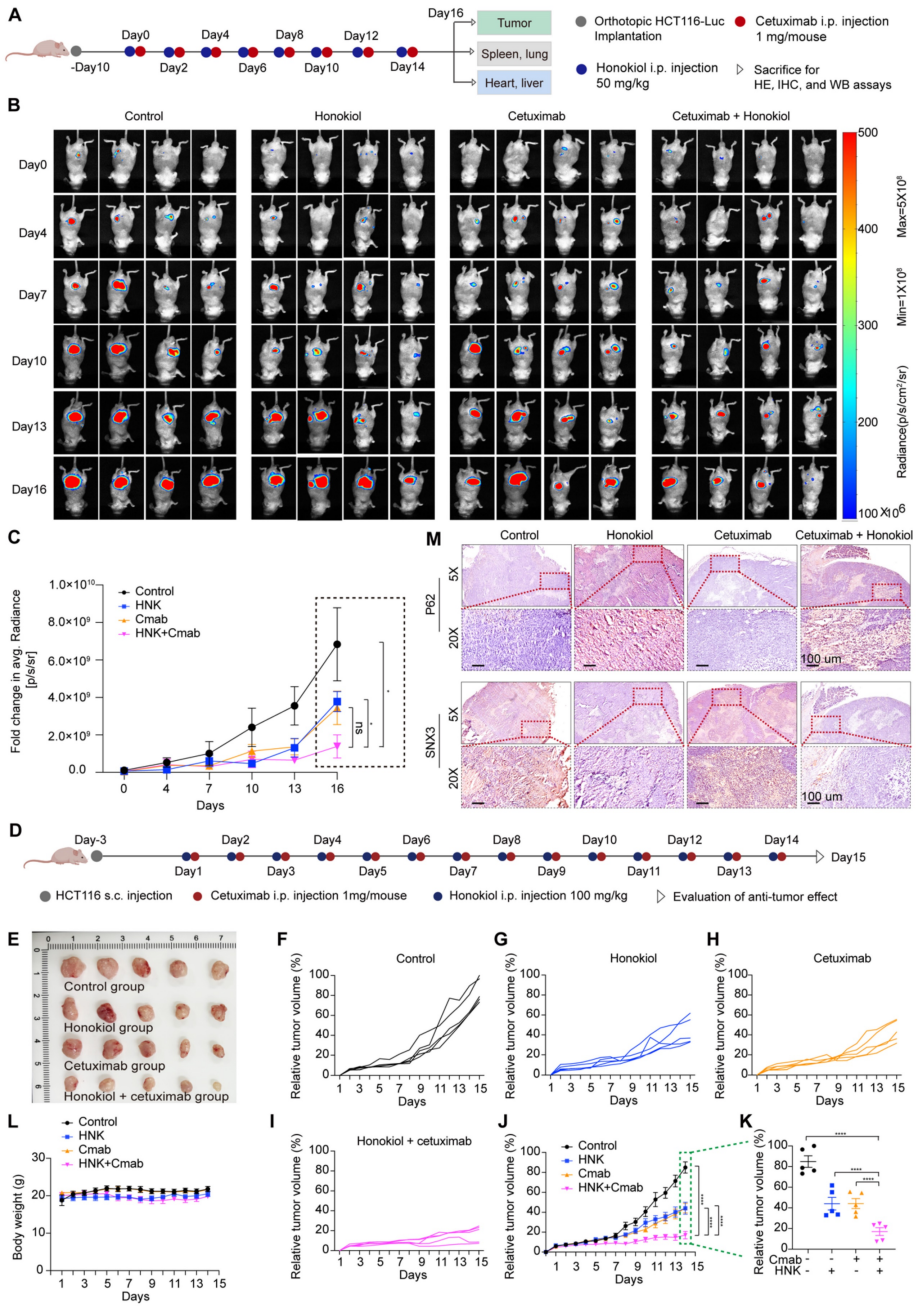
** Honokiol enhances the therapeutic efficacy of cetuximab in KRAS^G13D^ mutant CRC model. (A)** BALB/c nude mice were orthotopic implanted with 1 × 10^6^ HCT116-Luc cells, mice were administrated with 50 mg/kg honokiol and/or 1 mg/mouse cetuximab through intraperitoneal (i.p.) injection once every two days. **(B)** Bioluminescent imaging after orthotopic inoculation HCT116-Luc cells in mice with honokiol and/or cetuximab treated.** (C)** Fold changes in average radiance of mice at different timepoints after honokiol and/or cetuximab treated. Data are represented as mean ± S.E.M (n = 4), and significance was determined using two-tailed t test (* p < 0.05). **(D)** Scheme of tumor inoculation [subcutaneous (s.c.)] and treatment schedule in HCT116 tumor-bearing athymic nude mice. Three days after tumor inoculation, mice were treated with saline, 100 mg/kg honokiol, 1 mg/mouse cetuximab, 100 mg/kg honokiol + 1 mg/mouse cetuximab through i.p. once every day. **(E)** Photos of excised tumors from mice bearing HCT116 xenografts in different treatment groups on day 15 (n = 5). **(F-I)** Individual tumor growth kinetics in control (F), honokiol (G), cetuximab (H), honokiol + cetuximab group (I) (n = 5). **(J)** Average tumor growth kinetics for all treatment groups. Data are shown as mean ± S.E.M (n = 5), and significance was determined using two-way ANOVA test with Bonferroni multiple comparisons post-test (**** p < 0.0001). **(K)** Average tumor volumes at experimental endpoint (day 15) in all groups. Data shown as mean ± S.E.M (n = 5), and statistical significance was determined using two-tailed t test (**** p < 0.001).** (L)** Average body weight of HCT116 tumor-bearing mice over the course of therapy. Data are shown as mean ± S.E.M. (n = 5). **(M)** Immunohistochemical staining of p62 and SNX3 of orthotopic KRAS^G13D^ mutant tumor section.

To further evaluate the therapeutic efficacy of cetuximab in combination with honokiol *in vivo*, we initially established a KRAS^G13D^ orthotopic model of CRC by injecting luciferase-expressing HCT116 (HCT116-Luc) cells into the mesentery of the colon of immunodeficient nude mice. Tumor growth was monitored by detecting the average radiance of the tumor sites through bioluminescence imaging. Ten days later, mice were randomly divided into different groups and treated with saline, honokiol, cetuximab or honokiol + cetuximab every two days (**Figure 7A**). Honokiol and cetuximab were administrated by intraperitoneal (i.p.) injection. Bioluminescence imaging was performed on days 0, 4, 7, 10, 13, and 16. As shown in Figure 7B, the honokiol + cetuximab group exhibited significantly inhibitory effects compared to the saline (p < 0.05) and honokiol (p < 0.05) groups. However, there was no significant difference between the honokiol + cetuximab and cetuximab groups, possibly due to the small sample size and large within-group variability (**Figure 7B-C**). To further validate the optimal combination of honokiol and cetuximab, we performed HCT116-bearing xenografts mice model in immunocompromised athymic nude mice, 100 mg/kg honokiol and 1 mg/mouse cetuximab were i.p. injection everyday (**Figure 7D**). HCT116 tumor-bearing mice treated with saline showed the highest rapid tumor growth, whereas honokiol and cetuximab alone showed moderate antitumor activity. Co-treatment with honokiol and cetuximab greatly enhanced the therapeutic efficacy compared to the treatment with honokiol or cetuximab alone at the end point of this study (**Figure 7E-K**). We also calculated the combination index (CI) using a reported method 16 to assess whether there was a synergistic effect of the combination treatment. The CI value of honokiol + cetuximab group was 1.14, indicating a potent synergistic effect of honokiol in combination with cetuximab *in vivo*. No obvious change in body weight was observed in any groups (**Figure 7L**). Moreover, no significant toxicity was found in orthotropic KRAS^G13D^ mutant CRC model (**Figure S6A**).

To better understand the *in vivo* mechanisms underlying this antitumor effect, we tested SNX3 expression in orthotropic KRAS^G13D^ mutant tumor sections by western blot analysis, and the results showed that SNX3 protein expression were strongly detected in saline group, whereas inhibited in honokiol combined with cetuximab or not (**Figure S6B**). Furthermore, we performed immunohistochemistry analysis and confirmed the high expression of SNX3 in saline and cetuximab groups, along with low expression of p62, when compared to honokiol and honokiol + cetuximab groups in orthotopic KRAS^G13D^ mutant tumor sections (**Figure 7M**). These results indicate the inhibition of the autophagy pathway, consistent with the *in vitro* results. Overall, honokiol enhances the sensitivity of cetuximab in KRAS^G13D^ mutant colorectal cancer, resulting in a therapeutic efficacy of cotreatment *in vivo*.

## Discussion

In CRC, baseline pre-existing *KRAS* mutations provide intrinsic resistance to anti-EGFR treatment 17. Cetuximab is an FDA-approved agent for advanced CRC patients without RAS mutations 18. Notably, approximately 36% of patients with CRC harbor KRAS mutations 19, but not all patients with KRAS mutations are resistant to EGFR-targeted therapies 20. There are conflicting reports regarding the KRAS codon G13D mutation. Several retrospective studies have demonstrated that cetuximab confers clinical benefits to patients with KRAS^G13D^ mutated mCRC compared to patients harboring other KRAS mutations 21, 22. This observation underscores the ongoing need for combination therapies. Honokiol, a promising antitumor compound, has been shown to exert activity against numerous human cancer cell lines, including inhibiting cell proliferation, arresting the cell cycle, and inducing cell apoptosis 23, 24. These effects increase the sensitivity of tumor cells to chemotherapy, targeted therapy, and regulate tumor-associated immune responses 17, 25, 26. Additionally, the combination of HNK could improve the therapeutic efficacy of chemotherapeutic or targeted drugs 27, 28. Nevertheless, the effect of honokiol on the responsiveness of KRAS^G13D^ mutant CRC cells to cetuximab and the underlying mechanisms remain largely unclear. In this study, we describe a therapeutic efficacy, target identification, and mechanism of a novel combination of cetuximab and honokiol, exhibiting a promising synthetically lethality *in vitro* and *in vivo* KRAS^G13D^ mutant CRC model.

Honokiol is known to inhibit AKT, and several studies have shown that honokiol activates autophagy by inhibiting AKT-mTOR 29, 30, so we performed western blotting to determine the *p*AKT/AKT levels in HCT116 and LoVo cells. Notably, we found that 25 μM honokiol and 25 μM honokiol + 20 μg/ml cetuximab did not change the *p*AKT/AKT levels (**Figure S8**), speculating that 25 μM honokiol may not inhibit AKT in HCT116 and LoVo cells. In addition, the PI3K/AKT/mTOR signaling pathway plays a key regulatory role in autophagy, and the PI3K/AKT cell survival signaling pathway is one of the two upstream pathways that regulate mTOR. Inhibition of PI3K can greatly block the downstream signaling pathways AKT and mTOR. However, 25 μM honokiol seemed to downregulate the expression of PI3K in HCT116 cells and upregulate the expression of PI3K in LoVo cells (**Figure S8**). Taken together, we speculate that 25 μM honokiol-induced autophagy in KRAS^G13D^ mutant CRC cells may not be regulated by the PI3K/AKT/mTOR pathway.

To determine the change in autophagy and SNX3-retromer levels in other cell types after honokiol treatment, we conducted western blot assays on NCM460 (normal intestinal), HT29 (KRAS wild-type colorectal cancer), SW480 (KRAS^G12V^ mutant colorectal cancer), SW1990 (KRAS^G12D^ mutant pancreatic cancer), and H1975 (EGFR^L858R/T790M^ mutant lung cancer) cells. As shown in Figure S9A, the conversion rate from LC3I to LC3II increased, accompanied by a gradual increase in p62 protein in a concentration-dependent manner with honokiol treatment, indicating autophagy flux inhibition in SW480 and SW1990 cells. However, p62 levels remained stable or decreased in HT29, NCM460, and H1975 cells (**Figure S9A**). These results indicate that honokiol activates or does not affect autophagy in non-KRAS mutant cells while inhibiting it in KRAS mutant cells. Furthermore, honokiol did not affect SNX3 and VPS35 protein levels in NCM460 and HT29 cells but increased them specifically in SW480 cells in a concentration-dependent manner (**Figure S9B**), suggesting inconsistent changes in the SNX3-retromer among different KRAS type cells. Additionally, we tested SNX3 levels in various KRAS type cells via western analysis. The results showed the highest expression level of SNX3 protein in LoVo cells, with high expression also observed in HT29 and HCT116 cells (**Figure S9C**), indicating a high basal activity of SNX3 in these cells. Therefore, we conclude that honokiol inhibits autophagy specifically in KRAS mutant cells while either activating or not affecting autophagy in non-KRAS mutant cells. Additionally, honokiol impairs the SNX3-retromer pathway specifically in KRAS^G13D^ mutant CRC cells.

*KRAS* mutations are often accompanied by metabolic adaptations, including elevated levels of basal autophagy and constitutive activation of macropinocytosis (**Figure S7**) 31-33**.** Subsequently, macromolecules are transported into acidic lysosomes to obtain nutrients for tumor growth. Meanwhile, lysosomes are dynamic organelles that are primarily associated with the degradation of macromolecules from the endocytosis and phagocytosis pathways. Effective lysosomal proteolytic degradation of autophagic and macropinocytotic cargo becomes important for tumor cell survival. Retromer is an evolutionarily conserved endosomal coat-complex consisting of vacuolar protein sorting components VPS35, VPS26 and VPS29 that orchestrate the endosome-to-Golgi and plasma membrane sorting of numerous transmembrane receptors 34-36. Mannose-6 phosphate receptor (M6PR) and its retromer-dependent endosome-to-Golgi retrieval are essential for the transport of lysosomal enzymes, such as Cathepsin B 37-40. The retromer complex is responsible for the retrograde transport of CI-M6PR through engagement with the sorting signal within the cytosolic tail of CI-M6PR. M6PR mediates the transport of pro-Cathepsin B from the trans-Golgi network to the endo/lysosome. In the lysosomes, pro-Cathepsin B is further processed via autocatalysis into a mature two-chain form consisting of an N-terminal light chain and a C-terminal heavy chain 12. Our study showed the mature two-chain form of CTSB was significantly decreased after honokiol administrated (**Figure 5C-D**). Previous studies have reported the impairment of retromer induced the suppression of Cathepsin B proteolysis in multiple disease 41-43. Efficient lysosomal proteolysis is achieved through the action of various lysosomal enzymes. Most lysosomal enzymes are synthesized as immature proenzymes and subsequently activated through proteolytic processing when reaching the lysosome 44, 45. In the present study, we demonstrate that the dysfunction of retromer following honokiol administrated in cetuximab treated cells caused the depletion of catabolic enzymes, thereby failing to degrade macropinocytic and autophagic cargoes, and undigested contents.

Available evidence has shown the crosstalk between SNX3-retromer and membrane receptors 46, 47, such as the retromer complex is involved in EGFR retrograde transport from endosome to the plasma membrane 40. Moreover, SNX3 was shown to bind to EGFR using a biotin proximal labelling approach 48. Taken together, these findings suggest that SNX3 could be a therapeutic target in the combination of honokiol and cetuximab. Besides, KRAS^G13D^ mutation site has been suggested to rely more strongly on EGFR signaling when compared with other *KRAS* alleles 49-51, and we then investigated the effect of combination treatment on EGFR expression in KRAS^G13D^ mutant cells via western blot analysis. We found a time-dependent downregulation of EGFR in both HCT116 and LoVo cells following the combination treatment (**Figure 6A-B**). In contrast, over-expression of SNX3 increased the expression of EGFR and promoted the activation of *p*EGFR, RAS GTP, *p*MEK, and *p*ERK (**Figure 6D**). Nevertheless, the decreased EGFR association with the lysosomal compartment is consistent with previous observations that these cells have reduced lysosome proteolytic activities 33. Collectively, KRAS^G13D^ mutant cells are more strongly dependent on EGFR signaling for growth, and the combination treatment disrupts EGFR pathway by inhibiting SNX3, removing this pro-proliferative pathway.

Considering the safety of the drug combination, we assessed the IC_50_ values of cetuximab and honokiol individually on NCM460 cells. The results demonstrated that cetuximab is entirely safe for normal intestinal epithelial cells, and 20-25 μM HNK also exhibited safety to NCM460 cells, both when administered for 24 hours and 48 hours (**Figure S1A**). Moreover, we wonder whether the combination of honokiol and cetuximab causes organ toxicity in orthotopic CRC models. Hence, we performed hematoxylin and eosin stain analysis, and the results suggested that HNK and/or cetuximab did not induce damage to heart, kidney, liver, lung and kidney *in vivo* (**Figure S6A**).

In summary, this study reveals that the administration of HNK and cetuximab, as a therapeutic potential combination, shows better anti-cancer effects *in vitro* and *vivo*. Honokiol disrupted SNX3 retromer function, induced the inhibition of autophagy and macropinoctosis fluxes, along with the inhibitory effect in EGFR signaling pathway. Therefore, the combination of honokiol and cetuximab represents a promising approach to treating KRAS^G13D^ mutant CRC.

## Methods

### Cell lines and reagents

The human colorectal cancer cell lines HCT116 and LoVo cells were derived from cell bank/stem cell bank, Chinese Academy of Sciences. HCT116 and LoVo cells were cultured in IMDM and DMEM-F12 medium, respectively. All cell lines were grown in a humidified incubator at 37 °C with 5% CO2 level. Honokiol was purchased from Shanghai Yuanye Bio-Technology Co., Ltd. (Shanghai, China). Cetuximab (HY-P9905), rapamycin (HY-10219) and bafilomycin A1 (HY-100558) were purchased from MCE. EIPA (Cayman, 14406) was obtained from Cayman. The natural product compound library was obtained from TargetMol (L6300). Recombinant Human SNX3 protein (ab109970) was purchased from abcam.

### Plasmid and antibodies

Both negative control (vector), SNX3 over-expressed plasmids were designed by GenePharma. Lipofectamine™ RNAiMAX Transfection Reagent (ThermoFisher Scientific, 13778075) was obtained from ThermoFisher Scientific. The primary antibodies against were as follows: GAPDH (Cell Signaling, 5174,1:1000 for WB), β-actin (Cell Signaling, 4967, 1:1000 for WB), CTSB (Cell Signaling, 31718, 1:1000 for WB), CTSB (Santa Cruz, sc365558, 1:200 for IF), SNX3 (Novus, NBP3-20172, 1:200 for IHC), SNX3 (Abcam, ab56078, 1:1000 for WB, 1:200 for IF), p62 (Cell Signaling, 23214, 1:1000 for WB, 1:200 for IHC), EGFR (Santa Cruz, sc-373746, 1:200 for IF, 1:1000 for WB), pEGFR Try1068 (Cell Signaling, 11862, 1:1000 for WB), ERK1/2 (Cell Signaling, 137F5, 1:1000 for WB), pERK1/2 (Cell Signaling, 4370S, 1:1000 for WB), MEK1/2 (Cell Signaling, 9122S, 1:1000 for WB), pMEK1/2 (Cell Signaling, 9154, 1:1000 for WB), VPS26A (Abclonal, a14265), VPS29 (Abclonal, a13098), VPS35 (Abclonal, a9278). KRAS (Santa cruz, F234, 1:1000 for WB), and pan-RAS (Thermo Fisher Scientific, 1862335, 1:1000 for WB). The secondary antibodies against were as follows: Goat anti-Human IgG (H+L) Cross-Adsorbed Secondary Antibody, Alexa Fluor™ 647 Conjugate (Invitrogen, A-21445, 1;200 for IF), Anti-mouse IgG (H+L), F(ab')2 Fragment, Alexa Fluor® 488 Conjugate (Cell Signaling, 4408, 1;200 for IF); Anti-rabbit IgG (H+L), F(ab')2 Fragment, Alexa Fluor® 555 Conjugate (Cell Signaling, 4413, 1;200 for IF); SignalStain® Boost IHC Detection Reagent (HRP, Rabbit) (Cell Signaling, 8114); Anti-mouse IgG, HRP-linked Antibody (Cell Signaling, 7076, 1;1000 for WB); Anti-rabbit IgG, HRP-linked Antibody (Cell Signaling, 7074, 1;1000 for WB).

### High-throughput drug screen

KRAS wild-type cells (HT29, HCT8, RKO), KRAS^G12D^ (LS180), KRAS^G12V^ (SW480), KRAS^G13D^ (HCT116) mutant cells, and normal intestinal epithelial cells (NCM460) were cultured in 96-well plate at a density of 5000 cells per well for 24 hours. 882 compounds were added to a 96-well plate after being validated and checked for purity. Then, drugs at a single concentration (20 μM) were arranged to each well and incubated at 37 °C, and 5% CO2 for 48 h following drugs treatment. Cell viability was tested by cell counting kit-8, and inhibition rates were calculated. The inhibition rate of natural product compounds across different KRAS type cells was conducted in R and performed with pheatmap package v1.0.12.

### Cell viability assay

Cell growth *in vitro* was detected by cell counting kit-8 (MA0218-5, Meilunbio) assay according to the manufacturer's instructions. The cell growth curve was based on the corresponding normalized values of OD450. The percent cell viability was calculated as a percentage of absorbance change before and after different treatments.

### Colony assay

An equal number of cells (1 × 10^3^ cells) from different groups, including HCT116 vector, HCT116 SNX3 over-expressed, LoVo vector, and LoVo SNX3 over-expressed cells were seeded into 6-well plates. Subsequently, these cells were incubated for 2-3 days and then co-administrated with 25 μM honokiol and 20 μg/ml cetuximab for additional 5-7 days. The medium was changed every 2 days. Finally, live cells were stained with crystal violet.

### Apoptosis assay

Cells were plated in six-well plate at a density of 2 × 10^5^ cells per well for 24 h. Then, the tumor cells were treated with honokiol and/or cetuximab for 24 h. For apoptosis assay, cells were harvested and resuspended in 200 μl of 1 × binding buffer and double-stained with Annexin-V FITC and PI (Yeasen, 40302ES60) for 15 min at room temperature in the dark. Results were analyzed using a CytoFLEX S Flow Cytometer (Beckman coulter).

### Dextran uptake assay

Cells were seeded into 12-chamber slides for 12-16 h before honokiol and/or cetuximab treated for 24h. Then cells were treated with TMR-dextran 70 kDa (Invitrogen, D1818, 1 mg/mL) for 0.5-1 h. Cells were washed three times with cold PBS and then fixed with 4% paraformaldehyde (Solarbio, P1110). After fixation, cells were mounted in mounting solution containing DAPI. Confocal laser scanning microscopy (Olympus, FV3000) were used for acquiring images.

### Biolayer interferometry

Commercialized human SNX3 protein (500 μg/ml) was mixed with biotin reagent solution and incubated for 1 h at room temperature, and then desalted by using desalting columns prior to the start of the experiment. Streptavidin (SA) Biosensors (Sartorius, Gottingen, Germany) were immersed in PBS for 10 min at room temperature before the experiments were performed. SNX3 in 96-well H-bottom plates were directly immobilized on the SA biosensor. Honokiol was diluted by PBST (containing 5% DMSO and 0.02% Tween 20) to the appropriate concentrations with the final volume of 200 μL per well. At the same time, an equal volume of PBST was added to wells and set as the control group. The baseline, association, and dissociation steps were operated for 60, 120 and 180 s, respectively. The data were acquired and analyzed by ForteBio Octet Data Acquisition and Data Analysis software (version 11.0).

### Molecular docking

The crystal structures of proteins were retrieved from Protein Data Bank (SNX3:2YPS). Docking process: Discovery Studio Client was used to perform dehydration and hydrogenation of proteins. Pyrx-0.8 and AutoDock Vina39 were used for molecular docking, and Pymol software for mapping.

### Western blot analysis

The treated cells were harvested and lysed with RIPA buffer (Beyotime, #P0013) containing 1 mM protease and phosphatase inhibitor (ThermoFisher Scientific, #78441). The protein supernatant was carefully collected and centrifuged at 12,000 g for 20 min at 4 °C, and the protein concentration was determined by using a BCA protein assay kit. After heat denaturation, the equal amount of protein from each sample was resolved on 10-12.5% SDS-PAGE gels, which were then electroblotted onto polyvinylidene difluoride membranes (Merck Millipore, 3010040001). After blocking with a 5% solution of nonfat powdered milk for 1 h, the membranes were washed three times with TBST and incubated overnight at 4^◦^C in the appropriate primary antibody with gentle shaking. After, the membranes were incubated with HRP-conjugated secondary antibodies for 2 h at room temperature. BIO-RAD imager was used to analyze protein expression.

### Cellular thermal shift assay (CETSA)

Cells were seeded in 6 cm dishes at a density of 8 × 10^5^ cells and allowed to adhere for 24 h. After 24 hours, the cells were collected and centrifuged at 300 × g for 5 min to remove supernatants. The cells were then suspended in 1 × PBS buffer before adding 0.5 ml of 1 × PBS buffer containing protease inhibitor and flash-frozen with liquid nitrogen. The cells went lysis through three cycles of freezing and thawing. The resulting cell lysates were incubated with 0.1% (v/v) DMSO and honokiol for 60 min on ice, then heated at various temperatures (50.0, 51.4, 53.8, 57.5, 62.0, and 65.9 ℃) for 10 min. Next, the lysates were boiled for 10 min in loading buffer and subjected to western blot analysis.

### RNA sequencing and bioinformatics analysis

Cells were plated in 6 cm dishes at a density of 8 × 10^5^ cells per well for 24 h. Then the cells were divided into four groups: the control group, the honokiol group, the cetuximab group, and the combination treatment group. Each group had three replicates. After treatment for 24 h, the total RNA of cells was prepared by TRIzol (Invitrogen, CA, USA). LC-BIO Technologies (Hangzhou) Co., LTD. performed the Illumina Novaseq6000 sequencing. Gene expression was quantified using FPKM, and genes differential expression analysis was performed by DESeq2 software between two different groups. The genes with the parameter of false discovery rate (FDR) below 0.05 and absolute fold change ≥ 2 were considered differentially expressed genes. Differentially expressed genes were then subjected to enrichment analysis of GO functions and KEGG pathways.

### RNA interference

RNA interference was conducted as previously described. HCT116 and LoVo cells were transfected with the siRNA oligonucleotide using Lipofectamine™ 3000 (Thermo Fisher Scientific, L3000001) at 50% confluence. The siRNAs were generated by Tsingke Biotechnology and sequences were as follows: si-SNX3: sense, GGCUGGAGCAGUUUAUAAA; anti-sense, GGCUGGAGCAGUUUAUAAA. Stealth siRNA negative control (siNC) duplexes with a similar GC content were used as controls.

### Quantitative real-time PCR analysis

Total RNA was isolated with TRIzol according to the manufacturer's instructions, and cDNA was synthesized with a TransScript kit (Vazyme, R312) according to the manufacturer's protocol. mRNA expression was analyzed with an SYBR Green PCR kit (Vazyme, Q711-02). The data were normalized to GAPDH and analyzed using the ΔΔCt method. The primers used are listed in Table S1.

### Immunohistochemistry analysis

Tumor sections were deparaffinized, rehydrated followed by boiled in citrate buffer (pH = 6.0) for antigen retrieval using a pressure cooker. Endogenous peroxidases were inactivated in 1% H_2_O_2_ solution for 10 min and tumor sections were incubated in the blocking solution (1 × PBS, 10% goat serum) for 30 min. Then, the sections were incubated overnight at 4 °C with anti-SNX3 (Novus, NBP3-20172, 1:200) and anti-p62 (Cell Signaling, 23214, 1:200), which were then washed and incubated with HRP-conjugated secondary antibody for 60 min at room temperature. Finally, the DAB staining was performed, and nuclei were counterstained with hematoxylin. The IHC or immunofluorescent images were captured using a Digital Slide Scanner (NanoZoomer S60).

### Immunofluorescence

HCT116 and LoVo cells were seeded on coverslips and allowed to adhere for 24 h prior to treatment with HNK and/or cetuximab. Then, cells were fixed in 4% paraformaldehyde in PBS for 100 min at room temperature and permeabilized with 0.1% Triton X-100 (Solarbio, T8200) in PBS for 10 min. Following permeabilization, cells were treated with block buffer (1 × PBS, 10% goat serum) for 1 hour. Cells were incubated with primary antibodies diluted in blocking buffer overnight at 4°C. Cells were washed three times with PBST (1 × PBS, 1% tween 20) for 10 min, followed by incubation with Alexa Fluor-conjugated secondary antibody for 1 h at room temperature. Finally, the cells were washed three times with PBST again and stained with DAPI (1 μg/ml). Slides were examined by using a laser scanning confocal microscope (Olympus FV3000).

### Ras pull-down assay

Isolation of active GTP bound Ras was performed using the Active Ras Pull-Down and Detection Kit (ThermoFisher Scientific, 16117) following manufacturers protocol. Ras abundance was measured by western blot analysis. Western blot analysis of RBD pull-down lysates was performed with mouse anti-KRAS antibody, mouse anti-pan-RAS antibody (ThermoFisher Scientific, 1862335) and mouse anti-β actin (Cell Signaling, 4967).

### Transmission electron microscopy

Cells were plated to achieve ~80% confluence two-days later and treated with cetuximab and/or honokiol for 24 hours. Cells were washed twice with PBS and fixed with 2.5% glutaraldehyde fixed overnight at 4 °C. Cells were washed once with 4% sucrose in PBS and stained with 2% osmium tetroxide for 1 h while rotating. Cells were re-washed then dehydrated with increasing concentrations of ethanol (70%, 90%, 95% and 100% for 10 min/concentration). Cells were treated with propylene oxide for 30 min, before incubation in a 1:1 solution of 100% propylene oxide: 100% resin for 4 h and infiltration for two days, before embedding in fresh resin and polymerization at 70 °C for 24 h. Sections of 50 nm thickness were mounted on mesh copper grids. Images were captured using a Transmission Electron Microscope (ThermoScientific Talos L120C).

### *In vivo* therapeutic study

All animal experiments were in accordance with institutional animal care guidelines and conducted according to the committee-approved protocols of the Hangzhou Normal University. For the orthotopic CRC mouse model, 1 × 10^6^ HCT116-Luc cells were injected into the mesentery of the colon of immunodeficient 6-week-old female nude mice. Ten days post-injection, mice were randomly separated into four groups and treated with saline, honokiol, cetuximab or honokiol + cetuximab every two days. Tumor growth was monitored by detecting the average radiance of the tumor sites through bioluminescence imaging (Biospace PhotonIMAGER^TM^ OPTIMA, 2021248900) on days 4, 7, 10, 13, and 16, respectively. After 16 days of treatment, all mice were euthanized, and the major organs and tumors were collected.

To establish the HCT116 xenograft tumor model, 1 × 10^7^ HCT116 colorectal cancer cells in 100 μl of PBS mixed with 100 μl of Matrigel (BD Biosciences) were implanted subcutaneously on the right flank of athymic nude mice. Mice were monitored for tumor growth everyday according to the animal protocol. The mice were randomly divided into four groups (n = 5), which received treatment with saline, 100 mg/kg honokiol, 1 mg/mouse cetuximab, and honokiol + cetuximab. The day that first treatment was designated as day 1. Tumor size was measured using a caliper every day from day 1 to day 15, and the average tumor volume (mm^3^) was calculated as: 4π/3 × (tumor length/2) × (tumor width/2)^2^. Relative tumor volume (%) was calculated and presented according to a reported method 52. The largest tumor volume from the mouse at the end of this study was defined as 100%. The body weights of all the mice were also recorded over this period. Combination index was calculated as: (mean relative tumor volume _HNK+Cmab_) / (mean relative tumor volume _HNK_) × (mean relative tumor volume _Cmab_), and a ratio of >1 indicates a synergistic effect, and a ratio of <1 indicates a less than additive effect 16.

### Statistical analysis

The statistical analyses performed are explicitly stated in the figure legend. Parametric analyses were used for normally distributed data; non-parametric analyses were used for non-normally distributed data. All statistical analyses were performed using Prism 8 software. Statistical significance was attributed for p < 0.05. Graphs were annotated with asterisk (*) to denote p-values as follows: *p < 0.05, **p < 0.01, ***p < 0.001 and ****p < 0.0001.

### Ethics statement

All animal experiments were in accordance with institutional animal care guidelines and conducted according to the committee-approved protocols of the Hangzhou Normal University (Animal ethics approval No. HSD-20230829-05).

## Supplementary Material

Supplementary figures and table.
