# Supplementary material for: Honokiol enhances the sensitivity of cetuximab in KRASG13D mutant colorectal cancer through destroying SNX3-retromer complex
Source: Theranostics. 2024 Aug 26;14(14):5443–60. doi: 10.7150/thno.97180 (PMC11413778; doi:10.7150/thno.97180)
Supplement: Supplementary file 1 — Supplementary figures and table. [file thnov14p5443s1.pdf]

Supplementary Information for

Honokiol enhances the sensitivity of cetuximab in $KRAS^{G13D}$ mutant colorectal cancer through destroying SNX3-retromer complex

Qianru Zhu[1,2,#], Ruonan Zhang[1,#], Xiaoqing Gu[1], Ziming Zhao[2], Quan Gao[1,2], Min Chen[2], Qibiao Wu[2,], Tian Xie[1,2,*], Xinbing Sui[1,2,3,*]*

[1] School of Pharmacy, Hangzhou Normal University, Hangzhou, 311121, Zhejiang, China.

[2] State Key Laboratory of Quality Research in Chinese Medicines, Faculty of Chinese Medicine, Macau University of Science and Technology, Macau, P.R. China.

[3] Department of Medical Oncology, the Affiliated Hospital of Hangzhou Normal University, Hangzhou Normal University, Hangzhou, Zhejiang 311121, China.

[#] These authors contributed equally to this work.

* Correspondence: hzzju@hznu.edu.cn (X.S.), xbs@hznu.edu.cn (T.X.), qbwu@must.edu.mo (Q.W.)

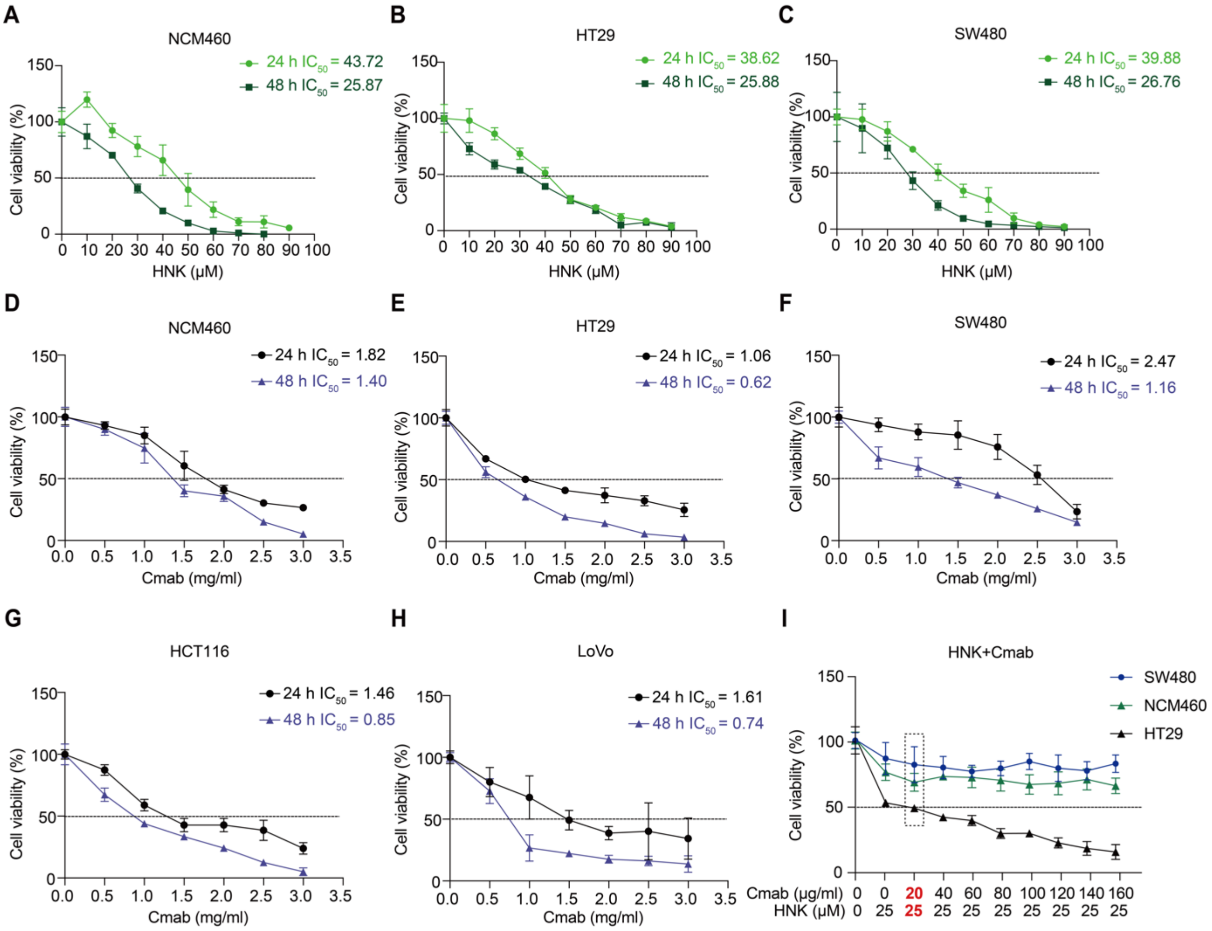

Figure S1 | The combination of honokiol and cetuximab to different type cell lines. (**A-C**) The cell viability of honokiol to NCM460, HT29, and SW480 cells for 24 and 48 h, respectively. (**D-H**) The cell viability of cetuximab to NCM460, HT29, SW480, LoVo, and HCT116 cells for 24 and 48 h, respectively. (**I**) The cell viability of 25 μM honokiol combined with different concentrations of cetuximab in SW480, HT29, and NCM460 cells for 24 h.

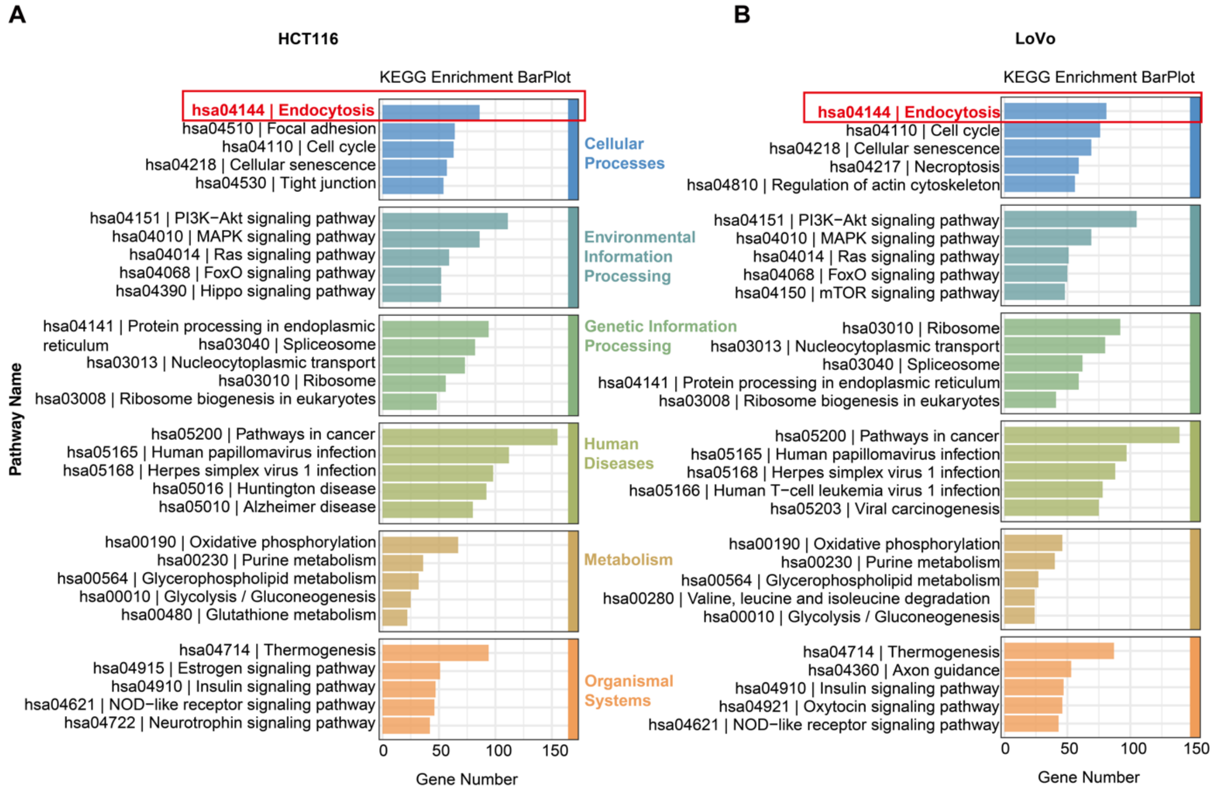

Figure S2 | KEGG analysis of HCT116 and LoVo cells following HNK and Cmab treatment. (**A-B**) KEGG analysis of HCT116 (A) and LoVo (B) cells among control, HNK, Cmab, and HNK + Cmab groups, and endocytosis were significant altered both in $KRAS^{G13D}$ mutant CRC cells.

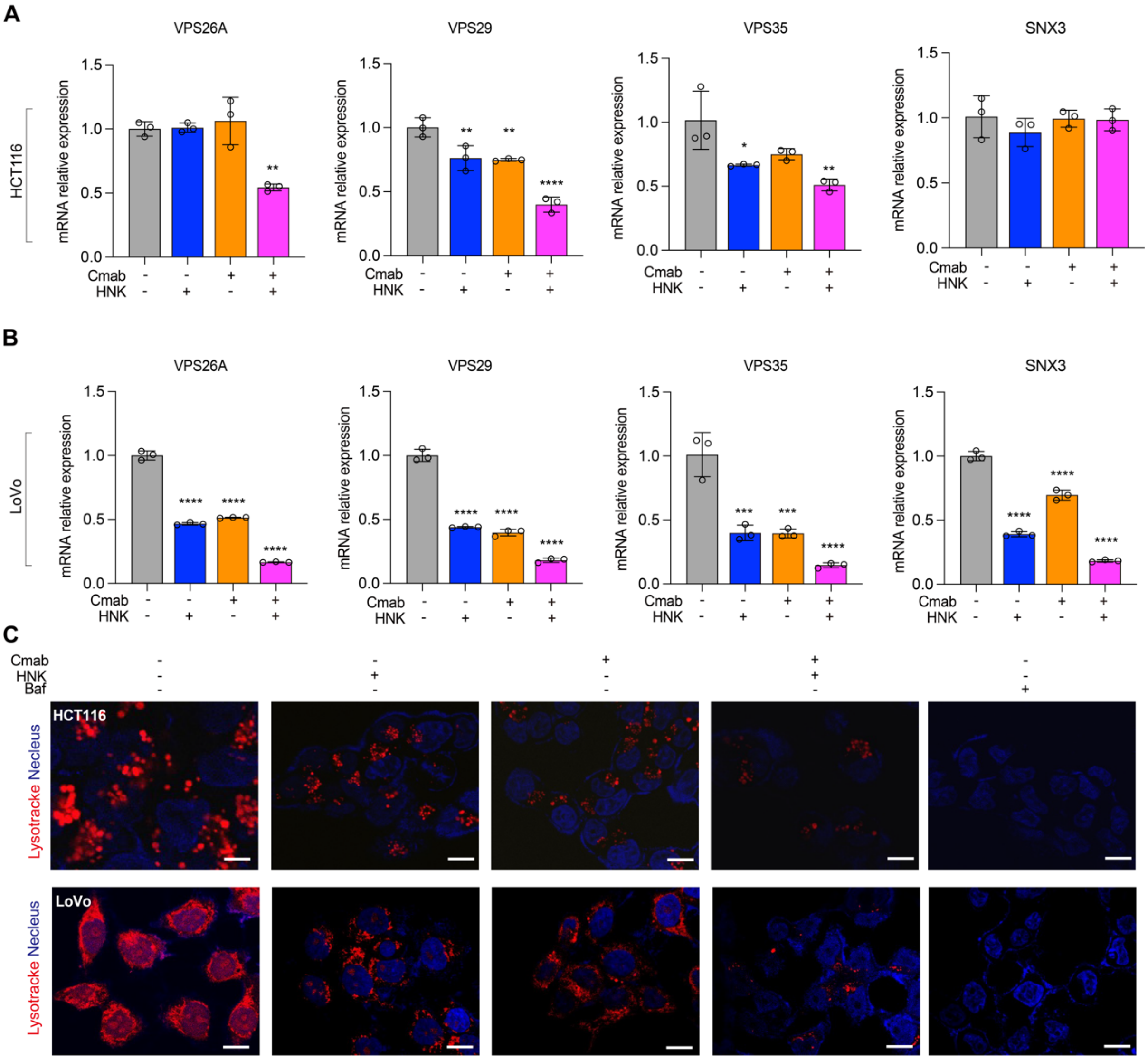

Figure S3 | Drug combination inhibits the function of SNX3-retromer and lysosomes. (A-B) The relative mRNA expression of SNX3 retromer (VPS29, VPS26, VPS35, and SNX3) in HCT116 (A) and LoVo (B) cells were quantified by qRT-PCR after the treatment with 25 μM HNK and 20 μg/ml Cmab for 24 h. $*p < 0.05$, $**p < 0.01$, $***p < 0.001$, and $****p < 0.0001$ by one-way ANOVA test with Dunnett's multiple comparisons test. **(C)** HCT116 and LoVo cells stained with lysotracker (red), a fluorescent dye labeling acidic, and nuclei were labeled with hoechst 33258 (blue). Scale bar represents 10 μm.

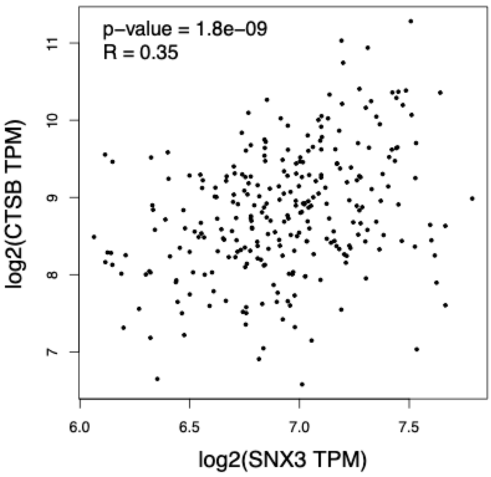

Figure S4 | Correlation analysis between *SNX3* and *CTSB* gene of CRC patients based on TCGA database.

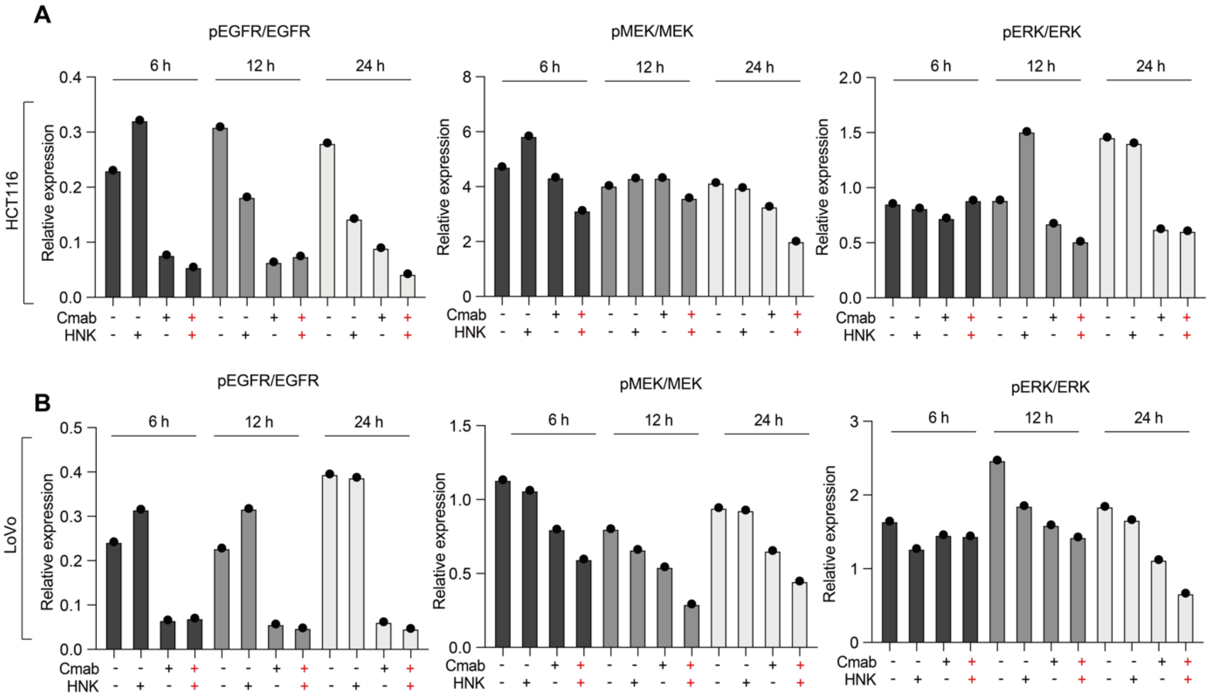

Figure S5 | Analysis of EGFR pathway in HCT116 and LoVo cells from treatment with honokiol and cetuximab. (A-B) Quantitaion of *p*EGFE/EGFR, *p*ERK/ERK, pMEK/MEK in HCT116 (A) and LoVo (B) cells after honokiol and cetuximab treated with 6 h, 12 h, and 24 h.

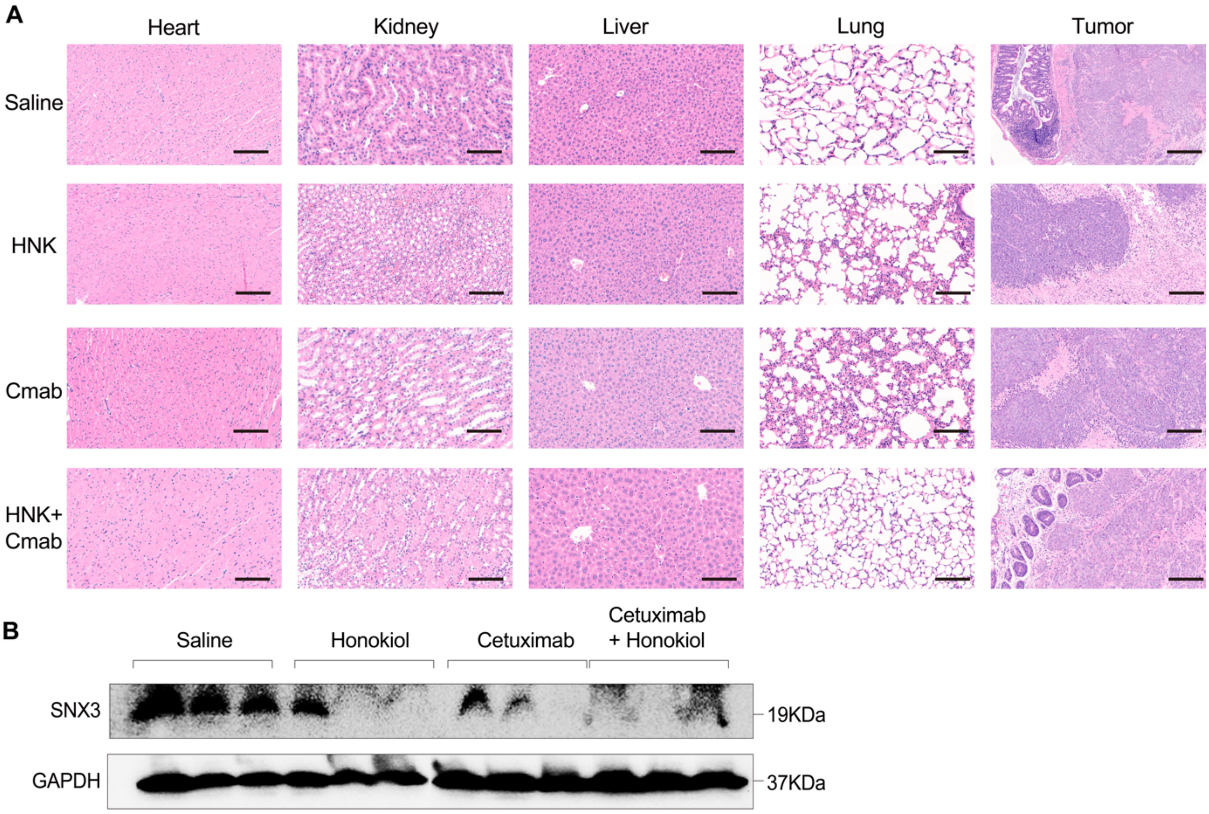

Figure S6 | Safety evaluation of honokiol and/or cetuximab *in vivo*. (**A**) Hematoxylin and eosin stain analysis of kidney, lung, liver, heart, and tumor section in orthotopic $KRAS^{G13D}$ mutant CRC mouse model. Scale bar represents 500 μm. (**B**) western blot analysis in tumor section after cetuximab and HNK treatment.

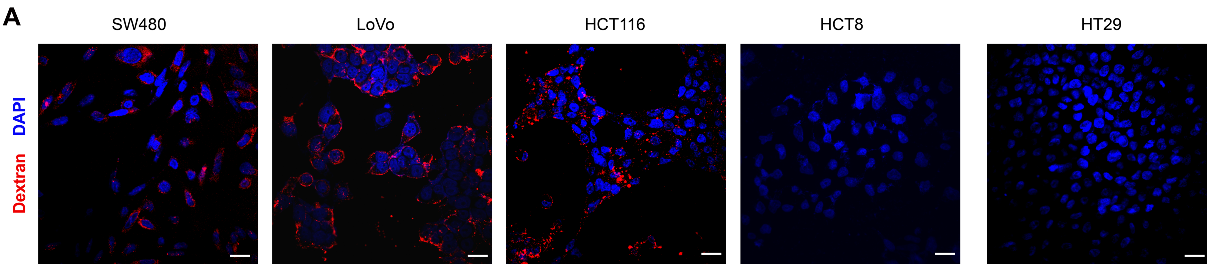

Figure S7 | Basal macropinocytosis levels in KRAS mutant CRC cells. (A) Represent images of micropinocytosis from KRAS mutant cells, including SW480 ($KRAS^{G12V}$), HCT116 ($KRAS^{G13D}$), LoVo ($KRAS^{G13D}$), and KRAS wild-type cells (HT29 and HCT8). Cells were labelled with 70 kDa tetramethylrhodamine dextran (TMR dextran), and nuclei were labeled with DAPI (blue), Scale bar represents 20 μm.

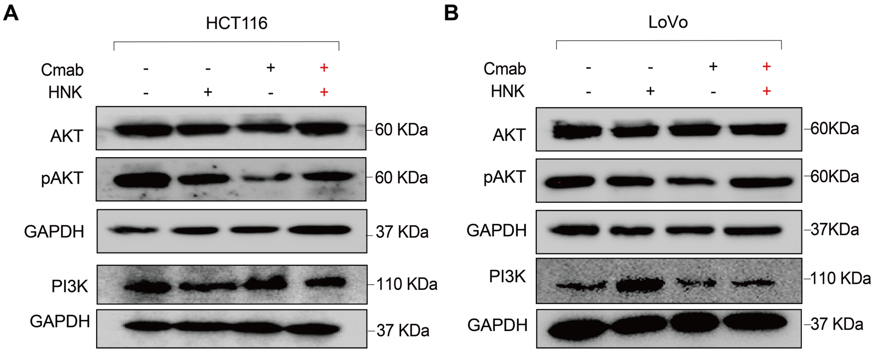

Figure S8 | Analysis of PI3K/AKT pathway in HCT116 and LoVo cells from treatment with honokiol and cetuximab. (A-B) the change of AKT, pAKT and PI3K protein levels in HCT116 (A) and LoVo (B) cells after honokiol and cetuximab treated for 24 h.

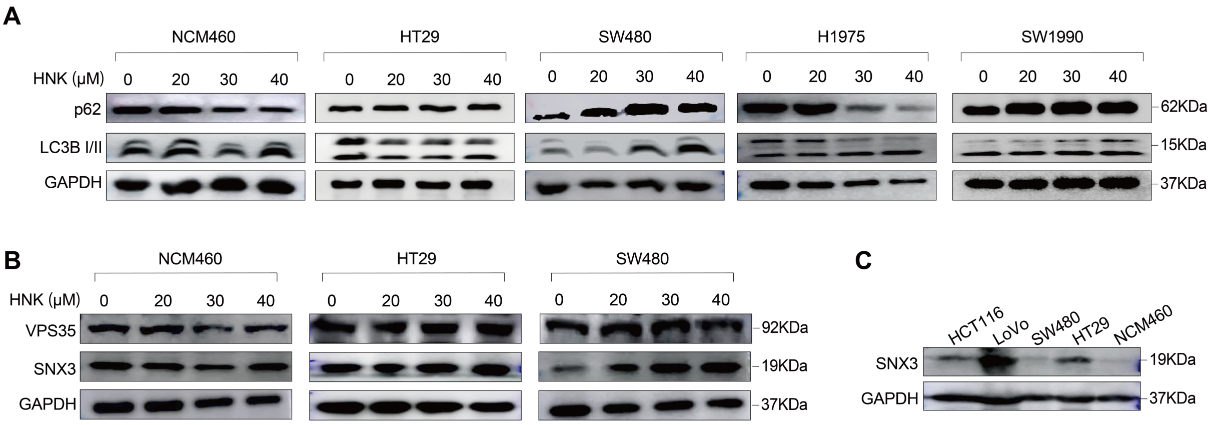

Figure S9 | Analysis biomarkers of autophagy and SNX3-retromer in multiple cell lines following honokiol treatment. (**A**) p62 and LC3B I/II protein levels in NCM460, HT29, SW480, H1975, and SW1990 cells after 0, 20, 30, 40 μM honokiol treated for 24 h. (**B**) SNX3 and VPS35 protein levels in NCM460, HT29 and SW480 cells after 0, 20, 30, 40 μM honokiol treated for 24 h. (**C**) Basel expression of SNX3 in various cell lines.

Table S1 | The sequence of primers used in this study.

Primers	Sequence
VPS35 forward	TGCTGATGAGCAGAGCCTTGTG
VPS35 reverse	CAGTGTGAAGCGAATCCGCTGA
VPS29 forward	CTCAAGACTCTGGCTGGTGATG
VPS29 reverse	CTGTCCAACAGTCACAACTTTCTG
VPS26A forward	GAGGCTAGAACACCAAGGAATTAG
VPS26A reverse	CTGCTCTGAGTCAGTTCTCCAG
GAPDH forward	GTCTCCTCTGACTTCAACAGCG
GAPDH reverse	ACCACCCTGTTGCTGTAGCCAA
